# A Non-Coronary, Peripheral Arterial Atherosclerotic Disease (Carotid, Renal, Lower Limb) in Elderly Patients—A Review PART II—Pharmacological Approach for Management of Elderly Patients with Peripheral Atherosclerotic Lesions outside Coronary Territory

**DOI:** 10.3390/jcm13051508

**Published:** 2024-03-05

**Authors:** Marcin Piechocki, Tadeusz Przewłocki, Piotr Pieniążek, Mariusz Trystuła, Jakub Podolec, Anna Kabłak-Ziembicka

**Affiliations:** 1 Department of Vascular and Endovascular Surgery, The St. John Paul II Hospital, Prądnicka 80, 31-202 Krakow, Poland; mpiech98@gmail.com (M.P.); tpkardio@kki.pl (P.P.); m.trystula@szpitaljp2.krakow.pl (M.T.); 2 Department of Cardiac and Vascular Diseases, Institute of Cardiology, Jagiellonian University Medical College, św. Anny 12, 31-007 Krakow, Poland; tadeuszprzewlocki@op.pl; 3 Department of Interventional Cardiology, The St. John Paul II Hospital, Prądnicka 80, 31-202 Krakow, Poland; jjpodolec@gmail.com; 4 Department of Interventional Cardiology, Institute of Cardiology, Jagiellonian University Medical College, św. Anny 12, 31-007 Krakow, Poland; 5 Noninvasive Cardiovascular Laboratory, The St. John Paul II Hospital, Prądnicka 80, 31-202 Krakow, Poland

**Keywords:** atherosclerosis, cardiovascular events, cardiovascular risk, carotid artery lesions, elderly patients, extra-coronary arterial disease, polypharmacy, prognostic factors, peripheral arterial disease, renal artery stenosis

## Abstract

Background: Aging is a key risk factor for atherosclerosis progression that is associated with increased incidence of ischemic events in supplied organs, including stroke, coronary events, limb ischemia, or renal failure. Cardiovascular disease is the leading cause of death and major disability in adults ≥ 75 years of age. Atherosclerotic occlusive disease affects everyday activity, quality of life, and it is associated with reduced life expectancy. As most multicenter randomized trials exclude elderly and very elderly patients, particularly those with severe comorbidities, physical or cognitive dysfunctions, frailty, or residence in a nursing home, there is insufficient data on the management of older patients presenting with atherosclerotic lesions outside coronary territory. This results in serious critical gaps in knowledge and a lack of guidance on the appropriate medical treatment. In addition, due to a variety of severe comorbidities in the elderly, the average daily number of pills taken by octogenarians exceeds nine. Polypharmacy frequently results in drug therapy problems related to interactions, drug toxicity, falls with injury, delirium, and non-adherence. Therefore, we have attempted to gather data on the medical treatment in patients with extra-cardiac atherosclerotic lesions indicating where there is some evidence of the management in elderly patients and where there are gaps in evidence-based medicine. Public PubMed databases were searched to review existing evidence on the effectiveness of lipid-lowering, antithrombotic, and new glucose-lowering medications in patients with extra-cardiac atherosclerotic occlusive disease.

## 1. Introduction

Aging is a key risk factor for atherosclerosis progression that is associated with increased incidence of ischemic events in supplied organs, including stroke, coronary events, limb ischemia, or renal failure [1,2]. Disseminated atherosclerosis of peripheral arteries, e.g., not limited to coronary arteries only, but including internal carotid artery stenosis (ICAS), renal artery stenosis (RAS), and lower limb peripheral arterial disease (PAD), is associated with a several-fold increase in cardiovascular risk events [3,4,5]. Atherosclerotic occlusive disease affects everyday activity, quality of life, and it is associated with reduced life expectancy [5,6]. In aging populations, cardiovascular disease is a leading cause of death and major disability in adults ≥ 75 years of age [6,7,8]. Although there is much evidence on the coronary artery disease management in the elderly, there is insufficient data on the management of older patients presenting with atherosclerotic lesions outside coronary territory.

Older people are the fastest growing age group in Europe. Data from the European Commission indicate that in January 2022, there were over 44.3 million people 75 years and older in 27 countries of the European Union, which accounts for 9.92% of all European citizens [9]. This number is expected to rise to one in five people by 2050 [10].

Regretfully, most multicenter randomized controlled trials (RCTs) exclude elderly and very elderly patients, particularly those with severe comorbidities, physical or cognitive dysfunctions, frailty, or residence in a nursing home [11]. This leads to insufficient data on the management of older patients presenting with diffuse atherosclerotic occlusive disease. Hence, due to a variety of severe comorbidities in elderly patients, the average daily number of pills taken by octogenarians can exceed nine [12,13,14]. Polypharmacy frequently results in drug therapy problems related to interactions, drug toxicity, falls with injury, delirium, and non-adherence. Therefore, there is a trend to deescalate medical treatment for cardiovascular disease and cardiovascular risk factors particularly in patients aged 80 years and older [15].

These gaps in the guidance on the appropriate medical treatment from evidence-based medicine on cardiovascular risk management in older patients, together with a variety of comorbidities requiring medical treatment, result in either reduction of cardiovascular drugs or their inappropriate use [16].

Therefore, we have attempted to gather data on the medical treatment in patients with extra-coronary atherosclerotic lesions, indicating where there is some evidence on the management in elderly patients and where there are gaps in evidence-based medicine.

## 2. Methods and Study Selection

A narrative review of all existing evidence on the effectiveness of lipid-lowering, antithrombotic, and new glucose-lowering medications in patients with extra-cardiac atherosclerotic occlusive disease was conducted. Public PubMed databases were searched using the following query: (‘peripheral arterial disease’ OR ‘PAD’ OR ‘renovascular disease’ OR ‘RVD’ OR ‘renal artery stenosis’ OR ‘RAS’ OR ‘carotid artery disease’ OR ‘ICAS’) AND (‘statins’ OR ‘PCSK9’ OR ‘ezetimibe’ OR ‘SGLT2’ OR ‘GLP-1’ OR ‘DDP-4’ OR ‘gliptin’ OR ‘flozin’ OR ‘glutide’ OR ‘aspirin’ OR ‘clopidogrel’ OR ‘ticagrelol’ OR ‘prasugrel’ OR ‘rivaroxaban’ OR ‘antithrombotic’ OR ‘antiplatelet’) AND (‘mortality’ OR ‘stroke’ OR ‘myocardial infarction’ OR ‘acute coronary syndrome’ OR ‘cerebral event’ OR ‘cardiovascular death’ OR ‘major adverse limb event’ OR ‘amputation’ OR ‘MACCE’ OR ‘MACE’ OR ‘MALE’ OR ‘heart failure’ OR ‘HF’ OR ‘CVD’). The occurrence of the above terms in the title or abstract was checked for all articles written in English between 1 January 1994, and 31 December 2023. A study was included in this narrative review if it fulfilled the following predefined inclusion criteria: (1) RCTs or prospective/retrospective observational studies or meta-analyses reporting on patients with and without administration of studied medication; (2) studies examining the comparisons of interest, reporting on short- and/or late-outcomes; (3) studies based on humans.

Endpoints of interest were all-cause and cardiovascular mortality, major adverse cardiac and cerebral events (MACCEs), hospitalization for heart failure (HF), PAD-related symptoms, major adverse limb events (MALEs), and amputation rates. Most studies evaluating mortality, MACCEs, MALEs, and symptomatic endpoints have shown the benefit of the analyzed medications in patients with extra-cardiac athero-occlusive disease.

Search results yielded 1698 articles published during this time interval. The results were filtered by article type, including RCTs, clinical trials, meta-analyses, observational studies, randomized controlled trials, and systematic reviews, which reduced the number of articles to 356.

Abstracts of these studies were reviewed to include studies with separate assessments of PAD, RAS, or ICAS groups and direct effects of treatment with lipid-lowering drugs, new glucose-lowering drugs, blood-pressure-lowering drugs, and antiplatelet or anticoagulant drugs. When duplicates were identified, the most recent study was included unless the earlier version reported more relevant outcomes. Most of the data have been abstracted from studies that mainly enrolled patients with CAD, intracranial disease, or atrial fibrillation. In addition, many studies reporting on stroke outcomes did not focus on the etiology of the primary cerebral event, resulting in limited evidence for ICAS. The above criteria allowed the identification of 51 studies that were included in the review. Finally, all identified studies were analyzed for the presence of evidence on the treatment efficacy and safety in elderly patients, particularly those 75 years of age and older. We searched for sub-analyses reporting outcomes in elderly patients, however, if we did not find distinct analyses, we reported this as a study limitation.

## 3. The Role of Pharmacotherapy in Atherosclerotic Arterial Disease Outside Coronary Territory

Cardiovascular risk factors accumulate with age [17,18,19,20,21,22]. In addition, there is an increasing incidence of diabetes, atrial fibrillation, renal failure, pulmonary disease, nervous system disease, gastrointestinal disease, and osteomuscular disease. Once identified, these comorbidities require additional pharmacotherapy. In the context of atherosclerosis, it is important to remember that some pharmacological interventions are more beneficial than others in stabilizing atherosclerosis and reducing the risk of acute ischemia. Substitution of drugs with poorly documented indications in the elderly by modern drugs with well-established benefits will avoid drug accumulation and overtreatment [23,24].

## 4. Pharmacological Interventions That Decelerate or Cause Regression of Atherosclerotic Lesions

Reducing atherosclerotic plaques is not an easy task. Our study showed that in a group of 466 patients after an acute ischemic incident who received full (guideline-directed) pharmacotherapy, a reduction or no increase in carotid plaque thickness was demonstrated in 37% of patients at 2 years of follow-up, and this percentage decreased to 26% at the end of 4 years of follow-up [25]. Attenuation of carotid atherosclerotic progression was associated with risk reduction of major cardiac and cerebral events (MACCEs) by 75% (hazard ratio, HR, 0.25; 95% confidence interval, CI, 0.15–0.42, *p* < 0.001) [25]. A meta-analysis by Willeit et al. of 119 clinical trials with a total of 100,667 participants found that even modest reductions in atherosclerotic progression (by 0.01, 0.02, 0.03, and 0.04 mm/year) reduced the risk of CVD by 16–37% [26].

Halting the progression of atherosclerotic lesions is the best indicator of treatment effectiveness [27,28,29,30,31]. Statins, angiotensin-converting enzyme inhibitors (ACEIs), angiotensin receptor blockers (ARBs), and calcium channel antagonists (amlodipine) have all been shown to slow the growth of atherosclerotic lesions [32,33,34,35,36]. There are doubts about the effects of modern antidiabetic drugs, like sodium–glucose cotransporter-2 inhibitors (SGLT2is), glucagon-like peptide-1 (GLP-1) analogues, dipeptidyl peptidase 4 (DDP-4) antagonists on endothelial function and the reduction in vascular stiffness, pulse wave velocity, and intima-media thickness [37]. However, the predominant opinion is that both GLP-1 analogues (to a greater extent) and SGLT2is (to a lesser extent) have beneficial effects on vascular compliance and function and are certainly more beneficial than sulfonylurea derivatives [37].

Antiplatelets and anticoagulants (aspirin, 2PY12 receptor inhibitors, cilostazol, rivaroxaban in a peripheral dose of 2.5 mg b.d., dipyridamole) do not reduce the thickness of atherosclerotic lesions; however, their effect is to limit thrombosis in ruptured atherosclerotic plaques and microvascular thrombosis, especially where collateral circulation is important, such as in PAD [38]. Natural antioxidants (curcumin, resveratrol, coenzyme Q10, bioflavonoids, lutein, β-carotene, vitamins A, E, and C, and the micronutrients selenium, zinc, manganese, magnesium, and many others) used as monotherapy do not show antiatherosclerotic effects [39,40]. On the other hand, in polytherapy, antiatherosclerotic effects and effects on the normalization of cardiovascular risk factors are small [41]. Caution should be taken when supplementing with vitamin D3. When present in the blood in therapeutic concentrations, vitamin D3 has beneficial antioxidant and metabolic effects [42,43]. Conversely, its deficiency exacerbates oxidative stress and its excess causes calcification of the intimal layer of the arteries and calcification of the atherosclerotic plaque matrix, which promotes the growth of atherosclerotic lesions and is associated with an increased risk of MACCEs [42,43].

Vasodilators that release endogenous or exogenous nitric oxide (e.g., arginine, nebivolol) do not affect the structure and size of the atherosclerotic lesions themselves [44]. Drugs such as dihydropyridine derivatives, for example, nitrendipine (a second-generation calcium channel blocker), act on the blood vessels mainly by reducing the smooth muscle tone of the vascular wall, which is particularly beneficial in the elderly. Nitrendipine inhibits the entry of calcium ions into smooth muscle cells in peripheral arterial vessels, but also into neurons (antagonizing the action of β-amyloid, the main protein responsible for Alzheimer’s disease) [45]. Thus, it contributes to improving cerebral circulation by protecting the blood–brain barrier and decreasing BP [45]. The randomized trial SYST-EUR showed that nitrendipine reduced the incidence of vascular dementia and dementia of mixed etiology by 55% (95% CI: 24–73%; *p* < 0.001) [46]. Additionally, it does not reduce renal blood flow. However, it should be used with caution in patients with aortic stenosis [45].

Drugs such as β-blockers, diuretics, α-blockers, and centrally acting drugs, although important in secondary prevention, treatment of hypertension, heart failure (HF), or arrhythmias, do not affect the size of atherosclerotic lesions but may worsen symptoms of stenoses in non-coronary arteries. β-blockers should be given if CAD is present. Exercise rehabilitation programs and cilostazol increase exercise time until intermittent claudication develops. Chelation therapy should be avoided [47,48,49].

Due to the limited data on the safety and efficacy of pharmacotherapy in patients over 65 years of age with associated carotid, renal, and lower limb atherosclerosis, many of these issues require additional multicenter and observational studies [50,51].

## 5. Pharmacotherapy in the Treatment of Atherosclerosis and Cardiovascular Risk Factors in Elderly Patients with Atherosclerosis in Extra-Coronary Territories

The goals of pharmacotherapy in patients with ICAS, RAS, and PAD include:

1. To reduce the risk of cardiovascular mortality and morbidity.
2. To reduce the risk of ischemic events typical for the location of the stenosis.
3. To reduce the symptoms of RAS (reduce the exacerbations of HF, angina, pulmonary edema) and PAD (especially in the presence of intermittent claudication).
4. To improve outcomes following revascularization (percutaneous or surgical).

### 5.1. Statins and Cholesterol-Lowering Drugs

#### 5.1.1. Primary Prevention in Elderly Patients

The treatment of elderly patients with statins arouses considerable emotions and is probably one of the most controversial issues [52,53,54]. On the one hand, some data show that in patients over 70 years of age with frailty syndrome (0–1 score on the Activities of Daily Living scale, ADL), non-use of statins increases mortality by 29% at a 4-year follow-up [53]. Conversely, findings from a systematic review of the literature led to the suggestion that lipid-lowering treatment is not appropriate for primary prevention in the elderly [54]. In the ALLHAT-LLT trial (2867 primary care participants), no benefit was found when pravastatin (40 mg o.d.) was given for primary prevention to older adults with moderate hyperlipidemia and hypertension, and a nonsignificant direction toward increased all-cause mortality with pravastatin was observed among adults 75 years and older [54]. The hazard ratios (HRs) for all-cause mortality in the pravastatin group vs. the control group were 1.18 (95% CI, 0.97–1.42; *p* = 0.09) for all adults 65 years and older, 1.08 (95% CI, 0.85–1.37; *p* = 0.55) for adults aged 65 to 74 years, and 1.34 (95% CI, 0.98–1.84; *p* = 0.07) for adults 75 years and older. Coronary heart disease event rates were also not significant [54].

In turn, statin therapy may be associated with a variety of musculoskeletal disorders, including myopathy, myalgias, muscle weakness, back conditions, injuries, and arthropathies [55,56]. These disorders may be particularly problematic in older people and may contribute to physical deconditioning and frailty. Statins have also been associated with cognitive dysfunction, which may further contribute to reduced functional status, risk of falls, and disability. This may be particularly true for seniors with frailty syndrome (≥7 points on the Frailty Assessment for Care planning Tool, FACT, and ≥8 points on the Clinical Frailty Scale), as no significant clinical benefit has been demonstrated in this patient group [52]. Initiation of treatment with statins or ezetimibe in these patients did not reduce the incidence of cardiovascular events, HF exacerbations, or strokes [52].

In line with this, a meta-analysis of trials conducted by the Cholesterol Treatment Trialists’ (CTT) Collaboration focused on the results of the PROSPER (primary and secondary prevention, pravastatin 40 mg) and JUPITER (primary prevention, rosuvastatin 20 mg) trials because they included a large group of elderly patients (PROSPER: *n* = 5804, mean age—75 years, and JUPITER: *n* = 5695, median age—74 years) [57,58]. Frailty and cognitive impairment were exclusion criteria in the PROSPER trial. The JUPITER study showed that for primary prevention, the number needed to treat (NNT) was 211 [52]. On the contrary, among 326,981 eligible veterans (mean age 81.1 years) in the US without atherosclerotic cardiovascular disease, 57,178 (17.5%) of subjects were started on statin treatment during the study period [59]. Statin use was associated with lower all-cause mortality (HR 0.75, 95% CI 0.74–0.76; *p* < 0.001), cardiovascular mortality (HR 0.80, 95% CI 0.78–0.81; *p* < 0.001), and MACCEs (HR 0.92, 95% CI 0.91–0.94; *p* < 0.001) with benefit evident within 2 years of statin treatment [59].

The analysis of the database of the Catalan primary care system (SIDIAP) including 46,864 people aged 75 years or more without clinically recognized atherosclerotic cardiovascular disease and diabetes showed that the HRs for new users of statins in 75–84 year olds were 0.94 (95% CI, 0.86–1.04) for atherosclerotic cardiovascular disease incidence and 0.98 (95% CI, 0.91–1.05) for all-cause mortality, and in those aged 85 and older they were 0.93 (95% CI, 0.82–1.06) and 0.97 (95% CI, 0.90–1.05), respectively [58]. However, in participants with diabetes, the HR of statin use in 75–84 year olds was 0.76 (0.65–0.89) for atherosclerotic cardiovascular disease and 0.84 (95% CI, 0.75–0.94) for all-cause mortality, and in those aged 85 and older they were 0.82 (95% CI, 0.53–1.26) and 1.05 (95% CI, 0.86–1.28), respectively [58]. In conclusion, this study demonstrated advantage of statin use in diabetic patients below 85 years of age and a lack of benefit in participants older than 74 years without type 2 diabetes and those with diabetes but aged 85 years and older.

Altogether, the combination of the multiple risks faced by the elderly and very elderly patients and the ALLHAT-LLT data showing that statin therapy in older adults may be associated with an increased mortality rate should be considered before prescribing or continuing statins for patients in this age category. This also results in frequent non-adherence to the prescribed statins and treatment discontinuation [60].

Because of conflicting data concerning statin use in the primary prevention of patients older than 70 years old, two RCTs (STAREE and PREVENTABLE) are currently underway to definitively demonstrate the effectiveness of statins for primary prevention of CV events as well as new dementia and disability in older adults [56]. The clinical trial of STAtin Therapy for Reducing Events in the Elderly (STAREE, NCT02099123) will examine whether treatment initiation with a statin (atorvastatin 40 mg) compared with placebo will prolong disability-free survival and reduce major cardiovascular events amongst healthy elderly people (≥70 years) with an estimated study completion in December 2025. However, high-risk primary care patients, including those with dementia, diabetes, and a total cholesterol level > 7.5 mmol/L, are excluded from this trial. The clinical trial of Pragmatic Evaluation of Events And Benefits of Lipid-lowering in Older Adults (PREVENTABLE, NCT04262206) will examine whether treatment with atorvastatin 40 mg o.d. vs. placebo could reduce the incidence of death, dementia, persistent disability, mild cognitive impairment, and cardiovascular events. The trial wants to recruit 20,000 community-dwelling adults 75 years of age or older without clinically evident cardiovascular disease, significant disability, or dementia, and follow them for up to 5 years (estimated median of 3.8 years). An estimated study completion is planned for December 2026.

#### 5.1.2. Secondary Prevention in Elderly Patients

The aforementioned dilemmas have resulted in undertreatment with statins and other non-statin lipid-lowering agents in elderly patients with established stable atherosclerotic disease [61]. In the study cohort including 24,651 patients older than 75 years with either coronary artery disease (CAD), cerebrovascular disease, or PAD, the prescriptions for moderate/high-intensity statin doses decreased with age. It is reported that 45% of adults over 75 received moderate/high-intensity statins in 2018. Statin prescription rates remained significantly lower for patients >75 years old as compared with those 65–75 years old (OR, 0.87; 95% CI; 0.85–0.89, *p* < 0.001). Women (OR, 0.77; 95% CI, 0.74–0.80; *p* < 0.001), patients who had HF (OR, 0.69; 95% CI, 0.65–0.74; *p* < 0.001), those with dementia (OR, 0.88; 95% CI, 0.82–0.95; *p* = 0.001), and underweight patients (OR, 0.64; 95% CI, 0.57–0.73; *p* < 0.001) were less likely to receive moderate/high-intensity statins [61].

Hence, the situation is also confusing regarding secondary prevention. A meta-analysis by Afilalo et al. (*n* = 19,569, age range 65–82 years) showed that statins reduce coronary mortality by 30% (RR 0.70; 95% CI, 0.53–0.83), non-fatal myocardial infarction (MI) by 26% (RR 0.74; 95% CI, 0.60–0.89), need for coronary revascularization by 30% (RR 0.70; 95% CI 0.53–0.83), and risk of stroke by 25% (RR 0.75; 95% CI 0.56–0.94) at a 5-year follow-up [62]. In this case, the NNT was 28.

Large trials mainly involve patients with cardiovascular risk factors and heart disease. Multicenter RCTs, such as the SPARCL randomized trial, which included patients with a recent stroke or TIA, are rare (Table 1) [63,64,65]. The SPARCL study enrolled 2249 patients aged ≥ 65 years (mean 72.4 years) and 2482 patients aged < 65 years who were treated with atorvastatin 80 mg [63]. Over a follow-up period of 4.9 years, younger patients showed a greater benefit from statin treatment than those over 65 years of age.

The study found a 26% reduction in the risk of stroke (HR 0.74; 95% CI 0.57–0.96; *p* = 0.02) in patients under 65 years of age and a 10% reduction in older patients (HR 0.90; 95% CI 0.73–1.11; *p* = 0.33). When analyzing only those aged 65 and over, there was a 21% reduction in the risk of stroke and TIA (HR 0.79; *p* = 0.01), a 32% reduction in coronary events (HR 0.68; *p* = 0.035), and a 45% reduction in the need for revascularization (HR 0.55; *p* = 0.0005) [63].

None of the above-mentioned studies evaluated statin treatment in the truly elderly (over 80 years), and the group of patients with atherosclerosis and stenosis of non-coronary arteries (carotid/vertebral, renal, lower limbs) was generally not analyzed. Moreover, patients having had cerebral ischemic episodes were a very heterogeneous group in terms of etiology. In the SPARCL study, 1007 patients had an average degree of internal carotid artery stenosis (ICAS) of 51% [64]. Following the introduction of atorvastatin at a dose of 80 mg, LDL cholesterol was reduced from a mean of 132 mg/dL to approximately 70 mg/dL. Statin treatment reduced the risk of stroke recurrence by 33% (*p* = 0.021), coronary events by 43% (*p* = 0.049), and the need for carotid revascularization by 56% at a 10-year follow-up (*p* = 0.006) [64]. A limitation in interpreting the results of this study is the relatively young age of the ICAS patients (mean 65.1 years) and the general lack of significant ICAS. In the Asymptomatic Carotid Surgery Trial (ACST), the risk of stroke in the asymptomatic ICAS group was 24.5% in patients without hypolipidemic treatment compared with 14.5% in those on cholesterol-lowering treatment [65].

In contrast, observational studies showed that a reduction in recurrent cerebral ischemic symptoms in patients with ICAS can be achieved with intensified pharmacotherapy, allowing a reassessment of the need for revascularization in these patients [66,67]. A multicenter observational study recruiting 387 patients with a neurologically symptomatic ICAS ≥ 50% showed a 7-day risk of recurrent stroke/TIA of 3.8% in patients who started intensive statin treatment on admission, compared to 13.2% in patients who did not receive a statin (90-day risk 8.9% vs. 20.8%, *p* = 0.01) [67]. However, the mean age in this population with ICAS > 50% was 71.1 (±10.6) years, hence, those over 80 years of age were a small group. Moreover, the same relationship was shown in those with ICAS who received antiplatelet treatment (*p* = 0.03), often using both drugs simultaneously. In the Lung et al. study, among 196 (median age 75 (Q1:Q3, 63–82) years) patients with stroke symptoms (Rankin score ≥ 3 points) due to critical ICAS > 70%, total cholesterol levels above 200 mg/dL were found in 117 (59.7%) patients and below 200 mg/dL in 79 (40.3%) patients [68]. At a 5-year follow-up, 25 (21.4%) deaths were observed in the group with cholesterol > 200 mg/dL compared with 28 (35.4%) in the group with low cholesterol (*p* < 0.05). The study authors found that low cholesterol was a risk factor for death (HR 1.88; 95% CI 1.09–3.23, *p* = 0.023); however, it is noteworthy that patients with cholesterol < 200 mg/dL were not receiving statin treatment at the time of discharge compared to patients with a baseline cholesterol > 200 mg/dL (11.4% vs. 72.6%, *p* < 0.001). Additionally, in this study, older age (HR 1.03, 95% CI 1.01–1.06, *p* = 0.012), chronic renal failure (HR 2.13 (1.18–3.5), *p* = 0.012), size of cerebral ischemic area (TACI, HR 3.28 (1.88–5.72), *p* < 0.001), and CAD (HR 1.91 (1.06–3.46), *p* = 0.032) were independent risk factors for death [68].

Similarly, as the indications for RAS revascularization in patients with renovascular hypertension diminish, the role of pharmacotherapy comes to the fore [69,70,71,72]. The pleiotropic effect of statins is particularly important in this group of patients, as it is associated with an 86% reduction in death (HR = 0.131; 95% CI: 0.039–0.438; *p* = 0.001) and protection of renal function (HR = 0.211; 95% CI: 0.070–0.637; *p* = 0.006), and it effectively prevents progression of RAS (RR 0.28; 95% CI: 0.10–0.77; *p* = 0.01) [69,72]. Statins are also highly relevant in patients over 65 years of age, as they halve the overall mortality, stroke rate, HF, and progression of renal failure to dialysis (RR 0.51; 95% CI: 0.47–0.57; *p* < 0.0001) [72].

Although lower limb arterial lesions are characterized by fewer inflammatory cells and lipids, patients with PAD who end up undergoing revascularization or amputation for vital indications have been found to have significantly lower rates of statin use compared to patients with stable PAD [72]. Additionally, in elderly patients with previously diagnosed PAD and hospitalized for major adverse limb events (MALEs), vascular and endovascular surgery centers report particularly low rates of patients treated with hypolipidemic and antiplatelet drugs [58,72]. According to data published by Ramos et al., there is an alarmingly high rate of statin discontinuation in patients over 80 years of age compared with younger patients (64% vs. 77%, *p* = 0.035), as well as the discontinuation of antiplatelet therapy (67% vs. 90%, *p* = 0.0001) [58].

A recent meta-analysis by Sagris et al. enrolling 39 studies and 275,670 patients with PAD showed that statin use was associated with a reduction in all cause-mortality by 42% (HR, 0.58; 95% CI, 0.49–0.67, *p* < 0.01), CVD by 43% (HR, 0.57; 95% CI, 0.40–0.74, *p* < 0.01), MACCEs by 35% (HR, 0.65; 95% CI, 0.51–0.80, *p* < 0.01), MI rates by 41% (HR, 0.59; 95% CI, 0.33–0.86, *p* < 0.01), the risk of amputation by 35% (HR, 0.65; 95% CI, 0.41–0.89, *p* < 0.01), and loss of vessel patency by 46% (HR, 0.54; 95% CI, 0.34–0.74, *p* < 0.01) [73,74,75]. Among patients treated with statins, the high-intensity treatment group was associated with a reduction in all cause-mortality by 36% (HR, 0.64; 95% CI, 0.54–0.74, *p* < 0.01) compared to patients treated with low-intensity statins. Statin use was associated with an increase in amputation-free survival by 56% (HR, 0.44; 95% CI, 0.30–0.58, *p* < 0.01) [73]. Of the aforementioned thirty-nine observational studies, only three reported outcomes in older patients, with those with a mean age between 73 and 80 showing favorable effects of statin treatment on the all-cause mortality, CVD, MACCEs, and MI (Table 1) [76,77,78].

In addition, proprotein convertase subtilisin/kexin type 9 (PCSK9) inhibitors are emerging as an additional lipid-lowering therapy for patients with cardiovascular disease [79,80]. In a sub-analysis of the FOURIER trial, of a total of 27,654 participants up to the age of 85 years, 3642 patients had symptomatic PAD. Patients with PAD receiving evolocumab as additional therapy to statins had a significantly greater reduction in the incidence of MALEs (0.27% vs. 0.45%, *p* = 0.0093) and MACCEs (9.5% vs. 13.0%, *p* = 0.004) compared to patients on placebo [81]. The relative efficacy of evolocumab was consistent regardless of patient age [82].

Vascular surgery societies’ guidelines recommend statins in patients with asymptomatic and symptomatic PAD (class I-A), and the use of statins is also indicated to increase walking distance (class I-B) without age criteria [83,84]. The guidelines further recommend the use of PCSK9 inhibitors in patients in whom statins and/or ezetimibe cannot be used or in those who have an unsatisfactory response to statins (class I-A). Similarly, PCSK9 inhibitors should be considered in patients with ICAS > 50% and dyslipidemia (class of recommendation: IIa-C) [83].

Low-density lipoprotein cholesterol targets are defined according to risk groups based on the 2019 recommendations of the Cardiovascular Pharmacotherapy Section of the Polish Society of Cardiology and may be as low as 35 mg/dL (<0.9 mmol/L) [83]. This applies to patients after multiple cardiovascular events and/or revascularization in specific anatomical sites such as patients after percutaneous revascularization of the left main coronary artery and/or multivessel CAD, patients with generalized atherosclerosis or multiple arterial areas with additional risk factors, and progression of atherosclerotic cardiovascular disease in patients who have achieved and continuously maintained LDL-C < 55 mg/dL (<1.4 mmol/L) [83]. The general recommendation in patients with PAD is to reduce LDL-C to <1.8 mmol/L (70 mg/dL) or by ≥50% if initial LDL-C is 1.8–3.5 mmol/L (70–135 mg/dL) (class of recommendation: I-C) [83].

In summary, in patients diagnosed with advanced non-coronary atherosclerosis, age does not justify discontinuing or reducing the dose of lipid-lowering drugs. Even if a statin does not result in regression of atherosclerotic plaque, achieving the recommended LDL cholesterol concentration is associated with a reduction in the rate of atherosclerotic progression. In patients with good tolerance, statins should not be discontinued because they have pleiotropic effects and significantly reduce the need for revascularization in this age group [85]. In turn, the avoidance of revascularization avoids complications caused by medication required for interventional treatment [86,87,88].

**Table 1:** Statin treatment in clinical trials, observational studies, and meta-analyses to improve outcomes in patients with carotid or peripheral arterial atherosclerotic disease with particular focus on elderly patients.

Study	Medication/Comparator	Type of the Study (RCT, OS, Meta-Analysis	Study Design	Elderly Patients >75 y.o.	F-U Period	Main Findings	Outcomes	Remarks/Limitations
**Carotid artery atherosclerotic disease**								
SPARCL trial Sillesen H et al., 2008 [64]	Atorvastatin 80 mg vs. placebo	RCT	High-dose atorvastatin in 1007 patients with recently symptomatic ICAS (51 ± 26%), mean age 65.1 ± 0.32 y.	NR	Mean: 4.9 y.	High-dose atorvastatin reduced stroke by 33% (95% CI, 0.47–0.94, *p* = 0.02), major coronary events by 43% (95% CI, 0.32–1.00, *p* = 0.05), and carotid revascularization by 56% (95% CI, 0.24–0.79, *p* = 0.006) among patients with stroke/TIA with documented carotid stenosis	Atorvastatin was safe and well tolerated, reduced mean LDL cholesterol level from 132 mg/dL to 70 mg/dL. Study confirmed the efficacy of high-dose statin in reducing risk of MACCEs	Elderly—not reported
Merwick A et al., 2013 [67]	Statin vs. placebo	RCT	Multi-center study in 262 patients with carotid stenosis ≥ 50% receiving statin (*n* = 114) pretreatment at TIA onset, or no statin use (*n* = 148), mean age 71.1 ± 10.6 y.	NR	90 d.	Statin pretreatment was associated with reduced stroke risk in patients with carotid stenosis (OR for 90-day stroke, 0.37; CI, 0.17–0.82, *p* = 0.008)	Reduction in early stroke risk in carotid stenosis with TIA associated with statin treatment	In the carotid stenosis patients, 39.1% underwent carotid revascularization (35.8% CEA, 3.3% CAS) within median 7 d. Statins are not specified by dose
**Lower extremities peripheral arterial disease**								
Sagris M et al., 2022 [72]	Statin vs. placebo	Meta-analysis	Meta-analysis of 12 studies reporting data on MACCEs, with a total of 27,768 patients with PAD, including 13,242 (47.6%) statin users and 14,526 (52.4%) non-statin users	NR	Mean: 46.7 m.	Statin use was associated with a reduction in all-cause mortality by 42% (HR, 0.58; 95% CI, 0.49–0.67, *p* < 0.01), CVD by 43% (HR, 0.57; 95% CI, 0.40–0.74, *p* < 0.01), MACCEs by 35% (HR, 0.65; 95% CI, 0.51–0.80, *p* < 0.01), MI rates by 41% (HR, 0.59; 95% CI, 0.33–0.86, *p* < 0.01)	Statin therapy is associated with improved cardiovascular outcomes in patients with PAD	Data on patients’ age usually NR
Sagris M et al., 2022 [72]	Statin vs. placebo	Meta-analysis	Meta-analysis of six studies, including 122,912 patients with PAD. Studies reported amputation risk in statin-treated patients (*n* = 44,861 (36.4%)) vs. non-statin-treated patients (*n* = 78,051 (63.6%))	NR	Mean: 58.4 m.	The risk of amputation was reduced by 35% (HR, 0.65; 95% CI, 0.41–0.89, *p* < 0.01) in the statin group. Statin use was associated with an increase in amputation-free survival by 56% (HR, 0.44; 95% CI, 0.30–0.58, *p* < 0.01)	Statin therapy is associated with improved limb outcomes in patients with PAD	Data on patients’ age usually NR
Sagris M et al., 2022 [72]	High-intensity statin vs. low-intensity statin	Meta-analysis	Five studies including 5071 patients with PAD. Studies included 2441 statin users (48.2%) and 2630 non-statin users (51.8%)	NR	Mean: 58.4 m.	The high-intensity statin group demonstrated a reduction in all-cause mortality by 36% (HR, 0.64; 95% CI, 0.54–0.74, *p* < 0.01) compared to patients treated with low-intensity statins	There is a dose-dependent mortality benefit of statins for patients with PAD	Data on patients’ age usually NR
de Grijs D et al., 2018 [73] Henke PK et al., 2004 [74]	Statin vs. placebo	OS	12-month patency rates after PTA for PAD or surgical bypass. The studies included 543 patients, including 295 statin users (54.3%) and 248 non-statin users (45.7%)	NR	Mean: 24 m.	The pooled HR for statin treatment in preventing loss of vessel patency was 0.54 (95% CI: 0.34–0.74, *p* < 0.01)	Statins prolonged vessel patency after revascularization for lower extremity PAD	In de Grijs et al. study, mean age of study group was 75 y. In Henke et al. study, mean age of study group was 64 y.
Aronow WS et al., 2002 [75]	Statin vs. no statin	OS	In total, 660 patients, mean age 80 y.o. with PAD, including 318 on statins and 342 without statins	NR	Mean: 20.6 m.	Statin use was associated with lower risk of MI (HR: 0.44, 95% CI: 0.36–0.54; *p* < 0.0001)	Statin therapy is associated with lower risk of MI in patients with PAD	
Matsuo Y et al., 2019 [76]	Statin vs. no statin	OS	In total, 1219 patients, median age 73 (67,79) with PAD, including 635 on statins and 584 without statins	Half of patients over 73 y., quarter over 79 y.	Median: 73 m.	Statin use was associated with lower risk of all-cause mortality (HR, 0.26; 95% CI, 0.21–0.32; *p* < 0.001) and MACCEs (HR, 0.33; 95% CI, 0.28–0.39; *p* < 0.001)	Statin therapy is associated with lower risk of all-cause mortality and MACCEs in patients with PAD	
Vidula H et al., 2010 [77]	Statin vs. no statin	OS	In total, 579 patients, mean age 73 ± 8.5 y. with PAD, including 242 on statin treatment and 337 without statin	NR	Mean: 3.7 y.	Statin use was associated with lower risk of all-cause mortality (HR, 0.51; 95% CI, 0.30–0.86; *p* = 0.012) and CVD (HR, 0.36; 95% CI, 0.14–0.89; *p* = 0.027)	Statin therapy is associated with lower risk of all-cause mortality and CVD in patients with PAD	
FOURIER trial Bonaca MP et al., 2018 [80]	PCSK9i (evolocumab) vs. placebo in patients on statin treatment and LDL cholesterol > 70 mg/dL	RCT	Of a total of 27,564 patients with cardiovascular disease, mean age 63 y., 13.2% had established PAD	NR	Median 26 m.	Patients with PAD receiving evolocumab had significantly greater reduction in the incidence of MALEs (0.27% vs. 0.45%, *p* = 0.0093) and MACCEs (9.5% vs. 13.0%, *p* = 0.004) vs. patients with PAD on placebo	Adding evolocumab to statin treatment is associated with additional MACCE and MALE risk reduction. Among patients with PAD compared to those without PAD, there was a greater risk reduction (1.5% vs. 2.4%)	Relatively young age of study participants. No data on elderly patients

ACEi—angiotensin-converting-enzyme inhibitor; ARB—aldosterone receptor blocker; ASA—aspirin; CCBs—calcium channel blockers; CI—confidence interval; CHF—congestive heart failure; CVD—cardiovascular death; DAPT—dual antiplatelet therapy; GLP-1—glucagon-like peptide-1; HF—heart failure; HR—hazard ratio; ICAS—internal carotid artery stenosis; IQR—interquartile range; IS—ischemic stroke; LDL—low-density lipoprotein; LVEF—left ventricular ejection fraction; MACCE—major adverse cardiac and cerebral event; MI—myocardial infarction; NR—not reported; OR—odds ratio; OS—observational study; PAD—peripheral arterial disease; PCI—percutaneous coronary intervention; PCSK9i—proprotein convertase subtilisin-kexin type 9 inhibitor; PTA—percutaneous transluminal angioplasty; RAS—renal artery stenosis; RCT—randomized controlled trial; RVD—renovascular disease; SGLT2is—sodium–glucose cotransporter-2 inhibitors; UA—unstable angina; y.—years.

### 5.2. Antiplatelet and Anticoagulant Drugs

Patients with non-coronary artery atherosclerosis, especially when it causes more than 50% lumen reduction, may require antiplatelet and/or anticoagulant therapy [89,90,91,92,93]. Thrombocytes play a key role in the pathogenesis of atherothrombotic complications in the arterial system, and inhibition of their activation and aggregation is the primary mechanism for preventing adverse cardiovascular events. The primary antiplatelet agent remains acetylsalicylic acid (aspirin, ASA).

Opinions are divided on the recommendation of ASA in asymptomatic ICAS > 50% (Table 2). Results of the observational studies Asymptomatic Cervical Bruit Study (HR 0.99, 95% CI 0.67–1.46; *p* = 0.61) and Asymptomatic Carotid Emboli Study (HR 0.45, 95% CI 0.31–0.66; *p* < 0.001), in which the endpoint was MACCE risk reduction, are conflicting [89,90]. A meta-analysis of 17 observational studies showed a benefit from the use of ASA in patients with asymptomatic ICAS > 50%, resulting in a 12% reduction in vascular incidents, mainly coronary arteries (*p* = 0.0001) [91].

Because two-thirds of patients with ICAS have advanced CAD, the 2023 European Society for Vascular Surgery (ESVS) guidelines suggest that low-dose aspirin (75–325 mg/day) should be considered in patients with asymptomatic ICAS > 50% to reduce the risk of MACCEs and MI (Class IIa-C) [94]. In cases of ASA intolerance or aspirin-dependent asthma, consider clopidogrel at a dose of 75 mg/day (class IIa-C). If ASA or clopidogrel cannot be used, consider dipyridamole monotherapy at a dose of 200 mg twice daily (class IIa-C) [94].

In symptomatic ICAS, antiplatelet therapy is always recommended with class I-A [17,94]. In poststroke patients with a carotid/vertebral artery etiology, short-term dual antiplatelet therapy (ASA + clopidogrel) for 3 weeks followed by long-term clopidogrel monotherapy or ASA with dipyridamole should be considered to stabilize atherosclerotic lesions [94,95]. However, this strategy is not supported by the results of a post hoc analysis of the CHANCE and POINT trials in patients with confirmed ICAS [96,97]. These trials included 276 patients with ICAS ≥ 50%, aged 70.3 ± 11.1 years old presenting with minor stroke or TIA within 12 h since symptom onset, scheduled to clopidogrel 75 mg plus ASA 75 mg versus ASA alone. Recurrent ischemic stroke risk reduction by clopidogrel plus ASA was not significant compared to ASA alone (12.2% vs. 13.9% (HR, 0.88, 95% CI, 0.45–1.72, *p* = 0.703)), whereas ICAS ≥ 50% constituted a major risk factor for stroke occurrence per se.

After CAS, it is necessary to continue ASA (75–325 mg/day) and clopidogrel (75 mg/day) together for at least 4 weeks (I-B), then one of these drugs long term. Because of concerns about the use of antiplatelet therapy in the elderly, the risk of bleeding can be assessed using the PRECISE-DAPT scale, which has also been tested in non-coronary areas [98].

Results from observational and RCTs comparing ASA with clopidogrel (CAPRIE trial) indicate a superiority of clopidogrel over ASA in reducing the incidence of MACCEs and MALEs [99]. In the CAPRIE trial, 19,185 patients with atherosclerotic disease manifested as recent stroke or MI or symptomatic PAD were included [100]. Compared with ASA, clopidogrel resulted in a 7.9% relative risk reduction in a combined end point of vascular death, stroke, MI, or rehospitalization for ischemic events or bleeding (15.1% to 13.7% at 1 year; *p* = 0.011) [99]. However, elderly patients aged 75 years or older constituted 201 patients in the CAPRIE trial and therefore were underrepresented [99]. Furthermore, among the exclusion criteria were uncontrolled hypertension, severe comorbidity likely to limit a patient’s life expectancy to less than 3 y., and severe cerebral deficit likely to lead to a patient being bedridden or demented [99]. In line with this, a systematic review and meta-analysis of the efficacy and safety of dual antiplatelet therapy (DAPT) in 24,873 elderly (65 y.o. or older) patients for secondary stroke prevention showed that DAPT was superior to ASA monotherapy but appeared to be equivalent to clopidogrel monotherapy [100]. Meanwhile, DAPT was accompanied by an increased risk of bleeding [97]. In a large observational cohort study, Huang et al. reported that clopidogrel use was associated with significantly lower risk of recurrent acute ischemic stroke (HR, 0.89; 95% CI, 0.83–0.96; *p* = 0.002), composite cardiovascular events (HR, 0.88; 95% CI, 0.82 to 0.95; *p* < 0.001), intracranial hemorrhage (HR, 0.71; 95% CI, 0.56–0.90; *p* = 0.005), and composite major bleeding events (HR, 0.89; 95% CI, 0.80–0.99; *p* = 0.04) compared with aspirin use [101]. This study included a total of 15,045 patients aged 80 years or above, of whom 1979 used clopidogrel and 13,066 used ASA following hospitalization for primary acute ischemic stroke [101]. In consequence, it is proposed that clopidogrel monotherapy may be a better choice for elderly patients in secondary prevention of stroke.

Although guidelines differ, antiplatelet therapy is not routinely indicated in patients with asymptomatic PAD (RCTs: POPADAD and AAA) who are not already receiving antiplatelet therapy for other reasons (e.g., coronary heart disease) (European guidelines class III-A) [17,94,102]. In contrast, according to US guidelines, antiplatelet therapy should be considered in patients with asymptomatic PAD (with ABI ≤ 0.9) due to the generalized nature of atherosclerosis and high cardiovascular risk in these patients (class IIa-C recommendation in the 2016 AHA/ACC guidelines) [103].

Antiplatelet therapy is indicated in patients with symptomatic PAD or after revascularization (RCT: CLIPS, class I-A/C recommendations). Results from observational and randomized trials comparing ASA with clopidogrel in mortality (CAPRIE trial) indicate the superiority of clopidogrel over ASA in reducing the incidence of MACCEs and MALEs [104]. The ESC guidelines identify clopidogrel as the preferred agent for monotherapy in symptomatic PAD, but there is a weak ranking for this recommendation (class IIb-B). Other societies have no such recommendation.

It remains an open question whether it is worthwhile to add a ‘small’ anticoagulant to antiplatelet therapy. Results from the multicenter, randomized COMPASS trial showed that rivaroxaban at the vascular dose (2.5 mg twice daily) in combination with ASA (100 mg once daily) in patients with clinically stable ICAS or PAD significantly reduced the incidence of MACCEs, stroke, and MALEs, including amputations, compared with ASA alone, but at the expense of increased major bleeding [105,106,107]. However, conclusions from the COMPASS sub-analyses are somewhat different. In the group of patients limited to PAD with stable clinical symptoms of intermittent claudication, MACCEs occurred in 5.2% of patients treated with rivaroxaban 2 × 2.5 mg/day plus ASA 100 mg/day compared with 7.2% of patients treated with ASA 100 mg/day alone (*p* < 0.05), with MACCEs in 1.8% vs. 3.4% (*p* < 0.05) and major bleeding in 3.3% vs. 1.9% (*p* < 0.05), respectively [83]. The risk of MALEs was greatest in patients with previous amputation or vascular intervention (3.8%), lower in patients with symptomatic PAD without previous amputation or vascular intervention (1.37%), and lowest in patients with asymptomatic PAD (0.5%). Thus, the benefit–risk ratio appears to be greatest in patients with previous amputation or vascular intervention and lower in patients with asymptomatic PAD. Due to the overall reduction in the risk of MACCEs in the COMPASS study, it was concluded that patients with coexisting CAD may benefit more from the addition of rivaroxaban, particularly following MI [105,106,107].

**Table 2:** Antiplatelet and antithrombotic medications used in clinical trials, observational studies, and meta-analyses to improve outcomes in patients with atherosclerotic carotid or peripheral arterial disease with particular focus on elderly patients.

Study	Antiplatelet, Anticoagulation Agents/Comparator	Type of the Study (RCT, OS, Meta-Analysis	Study Design	Elderly Patients ≥75 y.o.	F-U Period	Main Findings	Outcomes	Remarks/Limitations
**Carotid artery atherosclerotic disease**								
Hu X et al., 2022 [38]	ASA vs. placebo	Meta-analysis of 5 RCTs	Meta-analysis including 841 patients with established asymptomatic carotid atherosclerosis	NR	Mean: 2.55 y.	For cardio-, cerebrovascular events and all-cause death, there were no differences between the aspirin group (RR: 0.73, 95% CI: 0.41–1.31, *p* = 0.29) and the control group (RR, 0.88, 95% CI: 0.41–1.90, *p* = 0.74)	Aspirin did not alleviate the progression of carotid intima-media thickness compared with control patients	NR data on elderly patients, the mean age of study group around 64 y.
The Asymptomatic Carotid Emboli Study Markus HS et al., 2010 [89]	419 patients on ASA vs. 59 patients on placebo	Multicenter OS	474 asymptomatic patients with at least 70% asymptomatic carotid stenosis, mean age 71.5 ± 8.1 y.	NR	Mean: 2 y.	ASA significantly reduced risk of IS/TIA (HR 0.45, 95% CI, 0.31–0.66, *p* < 0.001) and risk of CVD/IS (HR, 0.13, 95% CI, 0.06–0.27, *p* < 0.001)	The risk of ipsilateral stroke after controlling for baseline antiplatelet therapy was 5.90 (1.68–20.72; *p* = 0.006) in patients with embolic signals compared to those without	NR data on elderly patients, although the mean age of study group was 71.5 y.
The Asymptomatic Cervical Bruit Study Côté R et al., 1995 [89]	188 patients on ASA 325 mg vs. 184 patients on placebo	RCT	372 asymptomatic patients with ≥50% asymptomatic carotid stenosis, mean age 65.9 ± 8.5 y.	NR	Median: 2.3 y.	The adjusted HR for ASA versus placebo for the primary outcome (the composite of TIA, stroke, MI, UA, or death) was 0.99 (95% CI, 0.67–1.46, *p* = 0.61); for vascular events only, the adjusted HR was 1.08 (95% CI, 0.72–1.62, *p* = 0.71)	ASA was found ineffective for the reduction of ischemic cardiovascular events or death from any cause in patients with asymptomatic carotid artery stenosis ≥ 50%	NR data on elderly patients
Antithrombotic Trialists’ Collaboration’s third systematic overview Baigent C et al., 2009 [91]	ASA 75–150 mg vs. placebo	Meta-analysis of 17 studies	Meta-analysis of 17 observational studies in patients with symptomatic and asymptomatic carotid stenosis	NR	NR	ASA reduced the risk of serious vascular events by 12% in patients with asymptomatic carotid stenosis (HR, 0.88, 95% CI, 0.75–0.93, *p* = 0.0001). ASA reduced the risk of serious vascular events by 17% in patients following TIA or IS (HR, 0.83, 95% CI, 0.75–0.93, *p* = 0.0008)	ASA use was associated with a reduction of vascular events, mainly coronary events; ASA showed a greater reduction of vascular events in secondary prevention patients following IS/TIA	NR data on elderly patients. Unknown number of patients with ICAS. The increase in risk of a major extracranial bleed with antiplatelet therapy (HR 1.6, 95% CI, 1.4–1.8)
The CHANCE trial Wang Y et al., 2013 [95]	ASA 75 mg plus clopidogrel 75 mg for 21 days vs. ASA 75 mg alone	RCT	Included 5170 patients with median age 62 y. (IQR: 55, 72) with minor IS or TIA within 24 h. after symptom onset, randomized to either ASA alone or ASA plus clopidogrel for 21 d.	≥65 y.o. 2141 patients	Median: 93 d.	In patients ≥ 65 y.o., during 90 d. of follow-up, DAPT reduced the occurrence of stroke by 29% compared to ASA alone (9.4% vs. 13.2% (HR, 0.70; 95% CI 0.54–0.90, *p* < 0.001). In a whole study group, there was non-significant increase in the rate of any bleeding events in DAPT (2.3%) vs. ASA group (1.6%) (HR 1.41; 95% CI 0.95–2.10, *p* = 0.09). The rate of hemorrhagic stroke was 0.3% in each group (*p* = 0.73)	Short-term dual antiplatelet treatment with ASA and clopidogrel reduces risk of stroke recurrence	NR data on the incidence of ICAS. No data on the bleeding rates, including the rate of hemorrhagic stroke in each group in patients aged ≥ 65 y.o.
The CHANCE trial Liu L et al., 2015 [96]	ASA 75 mg plus clopidogrel 75 mg for 21 days vs. ASA 75 mg alone	RCT	Overall, 1089 patients with MRA images available in CHANCE were included in this sub-analysis, including 608 patients (55.8%) with ICAS (median age 65.8, IQR 57, 73) and 481 (44.2%) without ICAS (median age 61, IQR 53, 70)	a quarter of patients ≥73 y.o.	Median: 93 d.	Patients with ICAS had higher rates of recurrent stroke (12.5% vs. 5.4%; *p* < 0.0001) at 90 d. than those without. In the ICAS group, there was no significant difference in stroke occurrence in DAPT group vs. aspirin alone (HR, 0.79, 95% CI, 0.47–1.32 vs. HR, 1.12, 95% CI, 0.56–2.25, *p* = 0.522), or the safety outcome of any bleeding event (interaction *p* = 0.277)	ICAS ≥ 50% was found to be an independent risk factor for stroke occurrence at 90 d. follow-up. Use of DAPT was not superior compared to ASA alone	No separate analysis for elderly with ICAS
POINT trial Yaghi S et al., 2021 [97]	ASA 75 mg plus clopidogrel 75 mg for 21 days vs. ASA 75 mg alone	RCT	Included 276 patients with ICAS ≥ 50%, aged 70.3 ± 11.1 y., presenting with minor stroke or TIA within 12 h since symptom onset	NR	90 d.	Recurrent ischemic stroke risk reduction by clopidogrel plus ASA was not significant compared to ASA alone (12.2% vs. 13.9% (HR, 0.88, 95% CI, 0.45–1.72, *p* = 0.703). When compared to patients with <50% carotid stenosis, ICAS ≥ 50% was an independent risk factor for IS occurrence in asymptomatic (HR 2.54, 95% CI 1.52–4.23, *p* < 0.001) and symptomatic ICAS ≥ 50% (HR 3.50, 95% CI 2.06–5.98, *p* < 0.001)	ICAS ≥ 50% was found to be an independent risk factor for stroke occurrence at 90 days of follow-up, despite DAPT vs. ASA alone use	No data on the bleeding rates
CAPRIE trial Bhatt DL et al., 2000 [99]	Clopidogrel 75 mg o.d. vs. ASA 325 mg o.d.	RCT	19,185 patients with mean age of 62.5 y. Efficacy of clopidogrel (75 mg o.d.) vs. ASA (325 mg o.d.) with ischemic stroke onset ≥ 1 week and ≤ 6 months before randomization	NR	Mean 1.91 y	For patients with stroke, the average event rate per year in the clopidogrel group was 7.15% compared with 7.71% in the ASA group, with an RR reduction of 7.3% (−5.7 to 18.7) in favor of clopidogrel (*p* = 0.26), however, it was non-significant.	Long-term administration of clopidogrel to patients with atherosclerotic vascular disease is more effective than ASA in reducing the combined risk of CVD/MI/IS. The overall safety profile of clopidogrel is at least as good as that of medium-dose ASA	NR on elderly patients
The COMPASS trial Anand SS et al., 2018 [98] Eikelboom JW et al., 2017 [106]	ASA 100 mg vs. ASA 100 mg/d + rivaroxaban 2.5 mg BID vs. rivaroxaban 5 mg BID	RCT	A total of 18,278 patients with stable atherosclerosis, mean age 68.2 y., including nearly 2000 patients with asymptomatic ICAS ≥ 50% or previous carotid revascularization	Age ≥ 75 y., 3821	Mean: 23 m.	Dual therapy with ASA and rivaroxaban reduced the MACCEs by 24% (HR, 0.76, 95% CI, 0.66–0.86, *p* < 0.001) in comparison with ASA alone in the whole group. Non-significant reduction occurred in the ICAS sub-group (HR 0.63, 95% CI, 0.38–1.05, *p* = 0.07). No difference was noted for asymptomatic ICAS (HR 0.85, 95% CI, 0.45–1.60, *p* = 0.61). Also, in the whole group, in patients above 75 y., the MACCE rate did not differ significantly between the two groups (6.3% vs. 7.0%; HR, 0.89, 95% CI, 0.69–1.14, *p* = 0.20). However, patients 75 y. and above had higher risk of bleeding on ASA + rivaroxaban compared to ASA alone (5.2% vs. 2.5%; HR, 2.12, 95% CI, 1.50–3.00, *p* < 0.001)	Evidence does not support use of a combination treatment with ASA plus low-dose rivaroxaban in patients with asymptomatic carotid stenosis	NR data on elderly in ICAS group. Not suitable in patients with atrial fibrillation or patients with IS during the last month or past hemorrhagic stroke or eGFR < 15/mL/min
**Lower extremities peripheral arterial disease**								
Antithrombotic Trialists’ Collaboration’s third systematic overview Baigent C et al., 2009 [91]		Meta-analysis of 42 trials	9214 patients with PAD			There was a proportional reduction of 23% (8%) in serious vascular events: CVD/MI/IS (*p* = 0.004), with similar benefits among patients with intermittent claudication, those having peripheral grafting, and those having peripheral angioplasty		
CAPRIE trial Bhatt DL et al., 2000 [99]	Clopidogrel 75 mg o.d. vs. ASA 325 mg o.d.	RCT	19,185 patients with mean age of 62.5 y. Efficacy of clopidogrel (75 mg o.d.) vs. ASA (325 mg o.d.) PAD, intermittent claudication and ABI ≤ 0.85, history of previous leg amputation, or revascularization	NR	Mean 1.91 y	For patients with PAD, the average risk of a composite event (CVD/MI/IS) per year in the clopidogrel group was 3.71% compared with 4.86% in the ASA group; a relative risk reduction of 23.8% percent (8.9 to 36.2) in favor of clopidogrel (*p* = 0.0028), without statistical difference in terms of safety	Long-term administration of clopidogrel to patients with atherosclerotic vascular disease is more effective than ASA in reducing the combined risk of CVD/MI/IS. The overall safety profile of clopidogrel is at least as good as that of medium-dose aspirin	
The Aspirin for Asymptomatic Atherosclerosis trial Fowkes FGR et al., 2010 [102]	ASA 100 mg o.d. vs. placebo	RCT	Scotland cohort of 3350 patients with a low ABI (<0.95), mean age 62 y.o. (range 50–75)	NR	Mean 8.2 y.	For primary event (fatal or non-fatal MI or stroke or revascularization) no difference was found between groups (HR, 1.03; 95% CI, 0.84–1.27), or for vascular events (HR, 1.00; 95% CI, 0.85–1.17), or all-cause mortality (HR, 0.95; 95% CI, 0.77–1.16)	ASA use was not associated with a reduction in cardiovascular event rates in patients with asymptomatic PAD. However, a trend to significance was noted for major hemorrhage requiring admission to hospital in the ASA group compared to the placebo group (HR, 1.71; 95% CI, 0.99–2.97)	Age limit ≤ 75 y.o.
The meta-analysis of the COMPASS and VOYAGER trials Anand SS et al., 2022 [107]	ASA 100 mg vs. ASA 100 mg/d + rivaroxaban 2.5 mg BID vs. rivaroxaban 5 mg BID	RCT	11,560 patients with stable asymptomatic and symptomatic PAD and immediately after peripheral revascularization. Median age: 68 (63, 74)	Half of patients ≥63 y.o., a quarter ≥ 74 y.o.	Mean 23 m.	The composite of CV death, MI, IS, acute limb ischemia, or major vascular amputation was reduced by 21% (HR 0.79; 95% CI, 0.65–0.95; *p* = 0.012) in low-dose rivaroxaban 2.5 mg BID plus ASA 100 mg/d compared to aspirin alone. Meanwhile, the risk of major bleeding was increased by 51% with low-dose rivaroxaban plus aspirin compared to aspirin alone (HR 1.51; 95% CI, 1.22–1.87; *p* = 0.0002)	The absolute risk reduction was 1.20%/year. In one year, treating 1000 PAD patients with low-dose rivaroxaban plus ASA compared to ASA alone prevents 12 MACCEs or limb events and causes 6 major bleeds, of which 1 is a fatal or critical organ bleed, and 0 intracranial bleeds were caused	Not suitable in patients with atrial fibrillation or patients with IS during the last month or past hemorrhagic stroke or eGFR < 15/mL/min. No safety data in HF NYHA III and IV or LVEF < 30% (study exclusion criteria)

In both non-stroke and poststroke patients, using a combination of rivaroxaban 2 × 2.5 mg + ASA 100 mg significantly reduced the incidence of new stroke compared with ASA alone in those aged 65–74 years (0.4% vs. 0.6%, HR 0.54; 95% CI 0.36–0.82) and those aged 75 years and over (0.6% vs. 1.2%, HR 0.48; 95% CI 0.29–0.79), but not in those aged under 65 years (0.7% vs. 0.8%, HR 0.80; 95% CI 0.47–1.35) [105,106,107]. This effect was particularly pronounced in patients with coexisting diabetes. In sub-analyses, rivaroxaban plus ASA was shown to reduce the risk of embolic (cardiac) strokes, but not those associated with ICAS (HR 0.85; 95% CI, 0.45–1.60; *p* = 0.61).

Therefore, there are currently no guidelines recommending the use of rivaroxaban with ASA in symptomatic ICAS. There is also no evidence for such treatment in asymptomatic ICAS.

However, it is important to note that the COMPASS study did not include patients over 80 years of age but demonstrated that the combination of rivaroxaban and ASA was associated with a greater incidence of bleeding (mainly from the gastrointestinal tract) in middle-aged patients. The risk of bleeding is even greater in the elderly. Studies have shown that even low doses of rivaroxaban have a stronger anticoagulant effect in elderly patients (median age 83 (75–87) years) compared to younger patients (median age 30 (26–38) years) [108]. This is due to a prolonged prothrombin time, reduced activity of coagulation factors II and X, and reduced thrombin activation.

Rivaroxaban at a dose of 2 × 2.5 mg daily is not an equivalent treatment in patients with non-valvular atrial fibrillation and should not be used in patients who have had a stroke within the last month, have a history of hemorrhagic stroke, or have an eGFR < 15/mL/min. There are no data available for patients with NYHA class III and IV HF and EF < 30% (study exclusion criterion).

In contrast, in cases of ICAS, RAS, or PAD in patients who require chronic anticoagulation (those with non-valvular atrial fibrillation or venous thrombosis), an anticoagulant is given at an age-appropriate dose according to relevant guidelines, without the addition of an antiplatelet drug. Atrial fibrillation patients over 65 years of age with coexisting non-coronary artery atherosclerosis receive a CHA2DS2-VASc score of 2. However, there is an ongoing dilemma in very elderly patients of how to treat octogenarians and nonagenarians [109]. An exception is 4 weeks after endovascular revascularization with stent implantation in a non-coronary artery, in which an anticoagulant and an antiplatelet drug (preferably clopidogrel) are used to reduce the risk of stent thrombosis [83].

To summarize, age is not a factor that modifies the indication for antiplatelet therapy in non-coronary atherosclerosis. In patients with symptomatic ICAS, RAS, or PAD, there is a benefit in using antiplatelet therapy (ASA, clopidogrel). In patients at high risk of bleeding, which is often in the elderly, a drug with a lower risk of bleeding (clopidogrel) may be chosen for treatment, while drugs such as ticagrelor should be avoided.

Particularly, as the results of the Examining Use of Ticagrelor in Peripheral Artery Disease (EUCLID) trial comparing clopidogrel and ticagrelor in almost 14 000 symptomatic PAD patients showed no difference in the rates of MACCEs (10.6% versus 10.8%) between the two agents [110,111].

There are no trials involving prasugrel in PAD and ICAS. In asymptomatic patients with ICAS and PAD, no significant benefit of antiplatelet treatment in terms of stroke and MALEs has been documented but should be considered due to the risk of MACCEs and MI. Rivaroxaban as an addition to ASA may be particularly beneficial in patients after MALEs and after amputation and revascularization in PAD; however, this has little effect on MALEs and MACCEs in asymptomatic PAD. Furthermore, rivaroxaban does not reduce the risk of stroke in patients with ICAS. When full anticoagulation is indicated, the addition of drugs from other anticoagulant classes is not recommended, except in situations directly related to peripheral artery revascularization, especially in combination with stent implantation. Triple anticoagulant therapy should be avoided, especially in patients over 70 years of age [112,113].

### 5.3. BP-Lowering Agents

BP control aims at reducing the risk of death and the development of cardiovascular disease, kidney disease, and dementia [114,115]. According to the guidelines of national consultants in family medicine, hypertensiology, and cardiology, the recommended BP during home measurements for individuals ≥ 65 years of age should not be greater than 135/85 mmHg, while for in-office measurements BP should not exceed 130–139 mmHg systolic and 70–79 mmHg diastolic. In contrast, new guidelines from June 2023 recommend office BP below <140/80 mmHg for the majority of patients due to favorable prognosis and organ protection. If the lower BPs are well tolerated, systolic pressures in the range of 120–130 mmHg and diastolic pressures of 70–80 mmHg are recommended in patients under the age of 79 years (recommendation classes IA and IB) [116]. However, for patients 80 years of age and older, the guidelines are more liberal. In patients without frailty syndrome, the first target for BP normalization is in the 140–150/80 mmHg in-office pressure range (recommendation class I-A). With good tolerance, a target systolic pressure of 130–139 mmHg (class II-B) should be considered. Since many patients over 80 are still somatically, mentally, and cognitively fit, lower systolic (120–129 mmHg) and diastolic (70–79 mmHg) pressures can be considered to provide further cardiovascular benefits [117]. BPs below 120 mmHg systolic and below 70 mmHg diastolic are not recommended (class III-C).

In patients with severe frailty syndrome and profound orthostatic hypotension, BP targets should be individualized (class I-C) [116]. Syncope and falls are the most frequently mentioned safety concerns related to antihypertensive treatment, but aggressive BP lowering may also negatively affect renal function, cognitive performance, quality of life, and survival [97]. A limitation of the ESC/ESH guidelines is due to the lack of randomized trials involving elderly patients with frailty syndrome and those aged 90 years or older (with and without frailty syndrome). Observational studies in this age group indicate a beneficial effect of normal BP and statin treatment [117,118]. According to the only two RCTs involving patients older than 80 years (HYVET, mean age 83 years) and a sub-analysis for patients ≥ 75 years (SPRINT), there were significant benefits of hypotensive treatment in reducing overall mortality and HF exacerbations [119,120]. In this patient population, mild–moderate frailty did not have a negative effect on treatment outcomes. In contrast, in patients with severe frailty syndrome (0-1-2 ADL score), ‘optimal’ BP (SBP < 130 mmHg) increases mortality [117].

The guidelines are highly relevant in the context of atherosclerosis and non-coronary artery stenosis. Several studies have shown that controlling systolic BP to <130 mmHg significantly reduces the progression of ischemic changes in the white matter of the brain and protects against dementia [121,122,123,124,125]. In particular, systolic BP > 160 mmHg strongly accelerates the progression of ICAS [126]. It is also remarkable that a post hoc analysis of two randomized trials (PreDIVA and SPRINT-MIND) showed that patients receiving ARBs, DHP calcium channel blockers, and thiazides/thiazide-like diuretics have less cognitive impairment compared to those treated with ACEis, non-DHP calcium channel blockers, and β-blockers [127,128].

In patients with PAD, the ALLHAT study results suggested an optimal systolic BP of 120–129 mmHg [129]. However, systolic BPs above 160 mmHg, as well as below 120 mmHg, and diastolic BPs below 60 mmHg were associated with an increased risk of ischemic events in the lower limb arteries such as PAD-related hospitalizations, necessary revascularizations, and deaths [129]. In the EUCLID study, systolic BP > 125 mmHg was associated with increased rates of MACCEs and MALEs, while a systolic BP < 125 mmHg was beneficial in PAD and reduced ischemic events in the lower limb arteries [130].

All classes of hypotensive drugs are used in the treatment of high BP among PAD patients. However, a combination of ACEis/ARBs and calcium channel blockers (amlodipine) is more important [83]. In PAD patients, ACEis or ARBs (class IIa recommendations) should be considered as first-line drugs. This preference for drugs that inhibit the RAA system is based on data from the Heart Outcomes Prevention Evaluation Trial (HOPE) and Ongoing Telmisartan Alone and in Combination with Ramipril Global Endpoint Trial (ONTARGET), in which the use of ramipril or telmisartan was associated with a reduction in cardiovascular risk in PAD patients but had no effect on limb ischemia outcomes or intermittent claudication walking capacity [131,132].

In patients with mild to moderate PAD, some β-blockers such as metoprolol or nebivolol (with vasodilatory effects) are also well tolerated, without worsening PAD symptoms or intermittent claudication walking capacity. The latter is included in a few studies involving elderly patients [133]. However, in a comparison of metoprolol and nebivolol in 128 patients with intermittent claudication and hypertension who had never been treated with a β-blocker before, the advantage of nebivolol was only found in terms of improvement of walking distance to pain onset [134]. The indications for β-blocker use in patients with PAD are based on coexisting diseases such as a history of MI or HF with impaired left ventricular systolic function (class IIa-B) [83].

Caution is advised in patients with MALEs, but even in patients with PAD treated with β-blockers, the results of observational studies were in no way worse than in patients not receiving these drugs. On the other hand, it is worth noting that some diuretics increase the risk of lower limb amputation, especially in patients with coexisting diabetes [134].

In conclusion, in elderly patients optimal SBP values should be not less than 120 mmHg and not higher than 150 mmHg, while DBP values should be not less than 70 mmHg and not higher than 85 mmHg depending on the somatic and functional status. At the same time, the optimal blood pressure values providing arterial homeostasis would be between 120 and 130 mmHg for SBP and between 70 and 80 mmHg for DBP.

#### Renovascular Disease

Atherosclerotic renovascular disease is the most common cause of ‘renovascular hypertension’ in the elderly. It should be suspected when previously well-controlled BP values become difficult to control in patients over 55 years of age or when renal function deteriorates [17]. In this clinical situation with high suspicion of RAS, renal Doppler ultrasonography, computed tomography, and/or magnetic resonance imaging of the kidney and renal arteries are recommended for the establishment of a RAS diagnosis [135,136,137]. With few exceptions, medical therapy with antihypertensive agents, antiplatelet drugs, and statins remains the cornerstone for management of patients with RAS.

Statins, ACEIs, and ARBs have a particular role in the pharmacological treatment of RAS patients (Table 3). Observational studies indicate a major role for RAA system blockade in reducing the risk of death (HR 0.61; 95% CI 0.40–0.91; *p* = 0.02) and in providing a better prognosis in unilateral RAS (HR = 0.24; 95% CI 0.08–0.71; *p* = 0.0098) [138,139,140]. RAA system inhibitors reduce renal interstitial damage, decrease proteinuria, and have a very important nephroprotective effect [138,139,140]. However, it should be noted that ACEis and ARBs can impair renal function on the stenotic side and, in the case of bilateral stenosis, cause severe and/or acute renal failure. Hence, monitoring of renal function is important when using ACEis and ARBs and they should be used with particular caution in patients with bilateral RAS or stenosis of the single functional kidney (recommendation class IIa) [116]. In addition, the use of calcium channel blockers was associated with a significant reduction in overall mortality (HR = 0.45; CI = 0.31–0.65; *p* ≤ 0.0001) as well as a reduction in CVD (HR = 0.51; CI = 0.29–0.90; *p* = 0.019) [141]. Statins slow atherosclerosis progression, and exert a nephroprotective effect, probably due to their pleiotropic properties [69,70,71,72]. Results from the Salford Renovascular Study showed that both antiplatelet agents and β-blockers have a beneficial effect in reducing the risk of patient death and that β-blockers also reduce the incidence of MI [142]. Optimally controlled diabetes (HbA1C < 7% or <53 mmol/mol) provides a nephroprotective effect through the reduction of adverse events after renal artery angioplasty (HR = 0.27; 95% CI = 0.13–0.57; *p* < 0.001) [143]. In contrast, a preoperative eGFR <45 mL/min/1.73 m^2^ (HR = 2.20; 95% CI = 1.20–4.04; *p* = 0.011) and a history of stroke (HR = 2.52; 95% CI = 1.19–5.34; *p* = 0.015) increase the risk of MACCEs and dialysis at a 2-year follow-up [143]. The role of modern oral antidiabetic drugs such as SGLT2 inhibitors, analogues of glucagon-like peptide 1 (GLP-1), and dipeptidyl peptidase 4 (DDP-4) inhibitors is still to be determined in patients with RAS [144].

**Table 3:** Medications used in patients with renal artery stenosis to improve cardiovascular and renal outcomes.

Study	Medication	Type of the Study	Study Design	Elderly Patients ≥75 y.o.	F-U Time Period	Main Findings
**Atherosclerotic renal artery stenosis**	NSAIDs					Medical agents associated with renal function deterioration, such as NSAIDs, should be avoided
Chrysochou C, et al. [138]	ACEis, ARBs	OS	621 subjects with RVD, mean age 71.3 ± 8.8 y., range 40–92 y.	NR	Median 3.1 y.	A major role of RAA system blockade in reducing the risk of death (HR 0.61; 95% CI, 0.40–0.91; *p* = 0.02)
Losito A, et al. [139]	ACEis	OS	195 patients with RVD, mean age 65.6 ± 11.2 y.	NR	Mean 4.5 y.	Reduced mortality associated with the use of ACEis (HR, 0.24; 95% CI, 0.08–0.71; *p* = 0.0098). An increase in serum creatinine level was associated with an increased mortality rate (HR 1.62; 95% CI, 1.04–1.28)
Hackam DG, et al. [140]	ACEis, ARBs	OS	3570 patients with RVD > 65 y.o. from province-wide health data in Ontario, Canada. Treatment group (*n* = 1877) mean age 74.3 ± 5.7 y.o., control group (*n* = 1693), 74.8 ± 5.8 y.o.	NR	Mean 2.0 y.	Patients receiving angiotensin inhibitors had a significantly lower risk for death or MACCEs (HR 0.70; 95% CI 0.59–0.82)
Deshmukh H, et al. [141]	CCBs vs. ACEi/ARB	OS	A total of 579 patients with RVD, mean age 76 ± 9.1 y.o.		Mean 3.5 y.	The use of CCBs was associated with a significant reduction in overall mortality (HR 0.45 (95% CI, 0.31–0.65); *p* < 0.0001) and CVD (HR 0.51 (95% CI, 0.29–0.90); *p* = 0.019). ACEis were associated with lower all-cause (HR, 0.60; 95% CI, 0.44–0.82, *p* = 0.0016) but not CVD mortality (HR, 0.73; 95% CI, 0.46–1.16, *p* = 0.19)
Cheung CM, et al. [69]	Statins	OS	79 patients who underwent renal angiography	NR	Mean 27.8 m.	Treatment with statin reduced the risk of RAS progression (RR 0.28; 95% CI 0.10–0.77) and increased a likelihood of RAS regression (HR 4.88; 95% CI 1.32–19.4)
De Silva R, et al. [70]	Statins	OS	Cohort study of 104 patients with RVD, mean age 65 y.o.	NR	Mean 2 y.	Statins use is associated with an 86% reduction in death (HR = 0.131; 95% CI: 0.039–0.438), protection of renal function (HR = 0.211; 95% CI: 0.070–0.637), *p* = 0.006)
Bates MC, et al. [71]	Statins	OS	Cohort study of 748 patients with RVD, mean age 70.7 ± 9.7 y.o., range 37–92 y.o.	NR	Mean 3.8 y.	Reduced mortality over 11-year follow-up with statins (HR 0.71; 95% CI 0.53–0.95)
Hackam DG, et al. [72]	Statins	OS	4040 patients with RVD > 65 y. from province-wide health data in Ontario, Canada. Statin group (*n* = 1682) mean age 74.1 ± 5.3 y.o., non-statin group (*n* = 2358), 74.9 ± 5.9 y.o.	NR	Median 3.3 (1.4; 5.0) y.	In patients over 65 years of age, statin use decreases rates of first cardiorenal event (MI, stroke, HF, acute renal failure, dialysis, or death) (HR, 0.51; 95% CI, 0.47–0.57; *p* < 0.0001)
Salford Renovascular Study Ritchie J, et al. [142]	ASA	OS	In total, 529 patients with RVD	NR	Median 3.8 y.	Antiplatelet agents (RR, 0.52; 95% CI, 0.31–0.89, *p* = 0.02) have a beneficial effect in reducing the risk of death
Salford Renovascular Study Ritchie J, et al. [142]	β-blockers	OS	In total, 529 patients with RVD	NR	Median 3.8 y.	β-blockers have a beneficial effect in reducing the risk of death (RR, 0.45; 95% CI = 0.21–0.97, *p* = 0.04) and the incidence of MI (RR, 0.74; 95% CI, 0.60–0.90, *p* = 0.003)
Badacz R, et al. [143]	Antidiabetics	OS	93 patients with T2DM and RVD and resistant hypertension, mean age 69.3 ± 7.2 y.o., range 47–84 y.o.	NR	Mean 2 y.	Optimally controlled diabetes (HbA1C < 7% (<53 mmol/mol) is associated with reduced incidence of major cardiac and cerebral events and renal replacement therapy following renal artery angioplasty (HR, 0.27; 95% CI, 0.13–0.57; *p* < 0.001)

ACEi—angiotensin-converting-enzyme inhibitor; ARB—aldosterone receptor blocker; ASA—aspirin; CCBs—calcium channel blockers; CI—confidence interval; CHF—congestive heart failure; CVD—cardiovascular death; DAPT—dual antiplatelet therapy; GLP-1—glucagon-like peptide-1; HF—heart failure; HR—hazard ratio; IQR—interquartile range; IS—ischemic stroke; LVEF—left ventricular ejection fraction; MACCE—major adverse cardiac and cerebral event; MI—myocardial infarction; NR—not reported; OS—observational study; PAD—peripheral arterial disease; PCI—percutaneous coronary intervention; RAS—renal artery stenosis; RVD—renovascular disease; SGLT2i—sodium–glucose cotransporter-2 inhibitor; UA—unstable angina.

### 5.4. Hypoglycemic Agents

The prevalence of type 2 diabetes is highest in older adults, in particular, and clinically relevant in reflecting the advancing age of the population with diabetes, in those aged ≥75 years [145]. In patients with non-coronary artery atherosclerosis and diabetes, strict blood glucose control and diabetes management according to mainstream principles are recommended [146]. The American Diabetes Association 2021 guidelines suggest a goal hemoglobin A1c of <7.0% for most patients with type 2 diabetes without significant hypoglycemia [147]. Glucose-lowering therapies with proven cardiovascular benefits are preferred, namely metformin, SGLT2is, and GLP-1 receptor agonists. Recently published American Heart Association/American College of Cardiology guidance on the use of these therapies for cardiovascular protection is available [148]. Metformin, the primary antidiabetic drug, continues to be an important medication in patients with PAD. However, new antidiabetic drugs are attracting more and more attention.

#### 5.4.1. Flozins (SGLT2 Inhibitors)

For example, SGLT2is have emerged as an important class of hypoglycemic drugs. For several years, an accompanying concern has been the reduction in cardiovascular risks [149]. Currently, the following SGLT2is are used: empagliflozin (10 mg/day, 25 mg/day), dapagliflozin (5 mg/day, 10 mg/day), canagliflozin (100 mg/day, 300 mg/day), and ertugliflozin (5 mg/day, 15 mg/day).

According to the ESC recommendations, which were based on the results of randomized multicenter trials, SGLT2is were included in class I-A recommendations in patients with or without type 2 diabetes, HF with reduced or preserved left ventricular systolic function (ejection fraction, EF < 40% and >45%), and in those with renal failure. These agents reduce the risk of CVD, reduce the need for hospitalization due to HF exacerbations, and have nephro- and cardioprotective effects [149]. When including SGLT2is as first-line treatment or in addition to metformin, it is important to ensure that the patient is treated with the maximum tolerated dose of a single RAA system inhibitor (ACEi or ARB) [150]. Glomerular filtration rate (eGFR) should be >20 mL/min/1.73 m^2^ for initiation of empagliflozin (EMPA-REG OUTCOME trial, EMPEROR-POOLED trial), >25 mL/min/1.73 m^2^ for dapagliflozin (DAPA-CKD trial, DECLARE-TIMI 58 trial), and >30 mL/min/1.73 m^2^ for canagliflozin (CREDENCE trial) [151,152,153,154,155,156,157]. The action of an SGLT2i is to reduce circulating blood preload and afterload, decrease glomerular filtration pressure, and block glucose reuptake in the proximal tubules of nephrons. This leads to an increase in urinary glucose excretion (approximately 80 g/day, 300 kcal/day), a reduction in BP, and a decrease in circulating blood volume [146]. A mild decrease in eGFR of 3–4 mL/min/1.73 m^2^ is generally observed after the first doses of an SGLT2i; however, if the decrease in eGFR exceeds 30% of the baseline value, the drug should be discontinued [150]. An increased urinary glucose excretion may result in an increase in urinary tract infections, including bacterial and fungal [151,152,153,154,155,156,157]. However, a meta-analysis of the RCTs showed similar incidence of urinary tract infections in patients receiving empagliflozin (RR 0.99, 95% CI 0.91–1.08) and canagliflozin (RR 1.10, 95% CI 0.90–1.33) as compared to placebo [151,152]. However, dapagliflozin showed a dose-dependent association evident with dapagliflozin 10 mg but not with dapagliflozin 5 mg daily [151,152].

The results for elderly patients with non-coronary artery atherosclerosis are highly controversial. Among those with a history of cardiovascular disease and hypertension, a meta-analysis of patients over 65 years of age, including 653 participants who received dapagliflozin and 535 receiving placebo, found no difference in cardiovascular event rates between groups (26 vs. 23 events, HR, 0.916; 95% CI, 0.512–1.640) [153]. However, the groups consisted of patients above 80 years of age only in a small part of the study. In the above-mentioned meta-analysis, the use of dapagliflozin in patients with ICAS, post-CAS, CEA, or stroke was not associated with differences in MACCE rates when compared to placebo. In contrast, a sub-analysis of the DECLARE-TIMI multicenter randomized trial involving 1025 patients with diabetes and PAD showed significant differences in the rates of MACCEs and MALEs in favor of those treated with dapagliflozin. Similar results were observed in a meta-analysis of EMPA-REG OUTCOMES, DECLARE-TIMI 58, and CREDENCE in which SGLT2 inhibitors were found to reduce MACCE outcomes in older adults (>65 years) by 17% (OR, 0.83; 95% CI, 0.70–0.99), numerically superior to the impact in younger individuals (OR, 0.94; 95% CI, 0.79–1.11) [153,154,155,156,157,158,159,160,161]. This led to an expert consensus statement on the management of older adults with type 2 diabetes and frailty [158].

The most controversial treatment is canagliflozin (Table 4) [154,159,160,161]. While reducing preload and afterload due to forced diuresis, SGLT2 inhibitors can cause hypovolemia and ‘thicken’ the blood [154]. This is most important in patients over 65 years of age, who often have coexisting dehydration caused by thirst. Hypovolemia promotes exacerbation of PAD, and both clinical situations coexisting in a patient may increase the risk of acute/critical lower limb ischemia [154]. Such a risk was identified in patients treated with canagliflozin in the CANVAS study, but not in the CREDENCE study [160,161,162]. Thus, it is possible that this was an effect of the high dose of canagliflozin (CANVAS: 300 mg/day) compared to 100 mg/day (CREDENCE), the age of the randomized patients (CANVAS: 63 years on average, 10 years older on average than patients included in trials with other SGLT2 inhibitors: 55 years), and coexisting vascular atherosclerosis (two-thirds of the group vs. approximately half of the participants in the CREDENCE study). In the CANVAS trial, a statistically significant increase in lower limb amputation due to acute limb ischemia was observed in patients treated with canagliflozin compared to placebo (6.3 vs. 3.4 per 1000 person-years [163]. This percentage was greater in patients with coexisting PAD but was also significant in those over 65 years of age without PAD. A meta-analysis of CANVAS and CREDENCE trials demonstrated that patients with PAD are at higher absolute risk for incidence of MACCEs, CVD, hospitalization for HF, and all-cause mortality compared with those without PAD [160]. However, canagliflozin reduced MACCEs (HR, 0.76; 95% CI, 0.62–0.92) without an increase in the relative risk of extended MALEs [164].

In the real world, the increased risk of amputation in patients over 65 years of age with PAD receiving canagliflozin has been confirmed in a group of nearly 311,000 patients [167]. This observational study compared treatment with canagliflozin and a GLP-1 analogue. The risk of amputation was 1.73 (95% CI: 1.30–2.29) in a group of patients over 65 years of age with a history of atherosclerosis of the coronary arteries, cephalic arteries, and PAD receiving canagliflozin compared with GLP-1 [167]. This represents 18 more amputations per 10,000 patients treated with canagliflozin for 6 months [161]. Insurance company records in the USA indicate that other SGLT2is may also be associated with a high risk of amputation, although not as often as canagliflozin [167]. Canagliflozin has been attributed to a particularly strong diuretic effect compared to other SGLT2is, with consequent hypovolemia, increased blood cell concentration, and increased blood viscosity [150]. Some diuretics also have similar effects with an increased risk of amputation. It has been suggested by nephrologists that SGLT2is should be avoided in bilateral RAS and stenosis of a single active renal artery [150]. Due to their mechanism of action on the glomerulus, a large decrease in eGFR (>20% of baseline) may be indicative of the presence of concomitant RAS, as is the case with bilateral RAS after the initiation of ACEis or ARBs [150].

#### 5.4.2. Glutides (GLP-1 Receptor Analogues)

GLP-1 receptor analogues are incretin drugs used in the treatment of type 2 diabetes that mimic the action of the incretin hormone GLP-1. They enhance insulin secretion in response to a carbohydrate-containing meal and prevent postprandial hyperglycemia [164,167,168,169]. In addition, they inhibit glucagon secretion by pancreatic beta cells and reduce hepatic glucose production, as well as delaying stomach discharge. GLP-1 receptor agonists include: liraglutide, dulaglutide, semaglutide, lixisenatide, and exenatide given subcutaneously or per os [165,166,168,169].

According to the results of RCTs, they appear to be the optimal drug to use in patients with type 2 diabetes or in elderly patients with PAD [165,166,168,169]. In a Danish observational study, in a group of 309,116 patients with type 2 diabetes patients receiving GLP-1 analogues, Schafer et al. noted a reduction in the risk of amputation compared to those without the treatment with an HR of 0.5 (95% CI, 0.54–0.74; *p* < 0.005), an effect dominated by liraglutide [166]. This risk reduction was consistent across different age groups. Out of 7333 total cases of amputation, 1849 (2.49%) amputations were performed in a group of 74,339 patients aged between 70 and 100 years old, compared to the amputation rate of 2.34% in younger patients (5484/234,777) [166]. It seems that GLP-1 analogues protect against foot or limb amputation, and they are less controversial (or more beneficial) compared to flozins [170,171].

A suggested mode of action of GLP-1 analogues is the anti-inflammatory effect through immune response modulation and is currently under investigation [172]. However, these drugs increase the risk of gastrointestinal disorders and bladder and gallbladder disease by 37%, cholecystitis by 36%, and urinary and/or gallbladder stones by 27% [166]. Lincoff et al. reported gastrointestinal disorders in 10.0% of patients in the semaglutide group and 2.0% in the placebo group (*p* < 0.001). Additionally, gallbladder-related disorders occurred in 2.8% and 2.3% of patients, respectively (*p* = 0.04) [173]. In line with this, in the SUSTAIN 6 trial, more patients discontinued active treatment because of adverse events, mainly gastrointestinal [174].

Also, it should be kept in mind that like in other studies with glucose-lowering medications, the coexistence of atherosclerotic occlusive disease with type 2 diabetes was associated with much higher incidence rates in patients with cardiovascular disease or PAD (10.01 ± 1.07 in SGLT2i users vs. 7.66 ± 1.58 in GLP-1 analogue users, *p* = 0.0269) compared to patients without these complications (2.07 ± 0.71 in SGLT2i users vs. 1.48 ± 0.53 in GLP-1 analogue users, *p* = 0.0155) [170]. However, the results are consistently in favor of GLP-1 receptor analogues.

#### 5.4.3. Gliptins (DDP-4 Inhibitors)

DDP-4 inhibitors block the enzyme that deactivates glucagon-like peptide-1 and increase meal-stimulated insulin secretion with low risk of weight gain and hypoglycemia [175,176]. By increasing GLP-1 availability, DPP-4 inhibitors promote insulin release and reduce postprandial glucose levels. However, adverse effects of DPP-4 include headaches and respiratory and urinary tract infections [159].

The results of randomized, observational, and cohort studies on the use of dipeptidyl peptidase 4 (DDP-4) inhibitors for diabetes monotherapy in patients ≥ 65 years of age are neutral or favorable regarding the rate of cardiovascular events [175,176,177,178,179,180]. In the TECOS (sitagliptin) and SAVOR-TIMI 53 (saxagliptin) trials, the use of gliptins in patients ≥ 65 years and/or ≥75 years of age was not associated with increased rates of MACCEs and unstable angina incidents [177,178]. In line with this, Johansen et al. found that in patients ≥ 65 years of age receiving linagliptin, fewer MACCE incidents and hospitalizations for HF exacerbations were observed compared to placebo (0.9% vs. 2.6%, *p* < 0.05) [179]. In contrast, in an observational study by Chang et al. involving patients ≥ 65 years of age, in which a DDP-4 inhibitor was used in combination with metformin compared to sulfonylurea derivatives with metformin, no advantage was found for either treatment regimen in terms of rates of CVD, MI, hospitalization for HF exacerbations, and stroke [180].

A severe drawback in the interpretation of the above-mentioned studies is that they practically have not involved patients with frailty syndrome, nor have they reported cardiovascular and limb outcomes for patients having a PAD, ICAS, or RAS. However, these issues are clinically relevant. Hypoglycemia is a major threat in elderly patients, particularly those with cognitive decline and frailty. These health conditions may result in late recognition of hypoglycemia and thus the inability to provide timely help, resulting in increased mortality rates [181,182].

Hypoglycemia was more frequently seen in patients on DDP-4. GLP-1 receptor agonists and SGLT2is do not cause hypoglycemia by themselves. GLP-1 receptor agonists act only in the presence of elevated glucose concentration, likewise SGLT2is reduce glucose absorption in the proximal tubule of the kidney, resulting in an increase in urinary glucose content and a reduction in HbA1c without hypoglycemic risk. However, a concomitant use of insulin or insulin secretagogues (glinides, sulfonylureas) may increase the risk of hypoglycemia [183].

With regard to DDP-4 use, in a study by Chen et al., of a total of 5145 type 2 diabetic patients with recent ischemic stroke, 1473 (28.6%) were 75 years of age or older [184]. In this study, the use of sitagliptin vs. other diabetic drugs showed no significant difference in the incidence of MACCEs and stroke in older vs. younger patients (12.1% vs. 11%, *p* = 0.463 and 8.6% vs. 8.0%, *p* = 0.705) [184]. Also, the incidences of pancreatitis and hypoglycemia in those receiving sitagliptin versus other diabetic agents were similar (0.2% vs. 0.2%, *p* = 0.838, and 1.9% vs. 2.0%; *p* = 0.730, respectively) [184].

Polyvascular disease was addressed by the sub-analysis of the SAVOR-TIMI trial [185]. In this RCT, of a total 16,492 type 2 diabetes patients receiving saxagliptin or placebo, 11,423 (69.3%) had established one-arterial-bed disease; 1298 (7.9%) had two-bed disease; and 104 (0.6%) had three-arterial-territory disease. Patients with arterial atherosclerotic occlusive disease were at higher absolute risk for MACCEs and all-cause mortality [185]. The adjusted risk for overall mortality increased in a similar stepwise fashion from 1.47 to 2.33 to 3.12 for patients with 1-, 2-, and 3-arterial-bed disease compared to patients with no established cardiovascular disease, respectively (trend *p* = 0.0001). However, saxagliptin use (as such) was not associated with increased risk of adverse outcomes compared to placebo across each additional diseased arterial territory [185].

#### 5.4.4. New Glucose-Lowering Medications—What Else Should Be Addressed

New antidiabetic medications entered clinical practice quite recently. The RCTs performed to introduce them on the market have several limitations. They include the panvascular character of atherothrombotic disease whose incidence is constantly growing due to its age-dependent specificity and the high prevalence of the major contributor—diabetes. Long-standing type 2 diabetes mellitus is inevitably associated with the development of macrovascular complications that in consequence lead to acute ischemic events, including coronary, cerebrovascular, and limb events [186,187,188,189]. Recent meta-analyses showed data on the incidence rates of lower limb amputation among patients treated with SGLT2is, GLP-1 receptor analogues, and DPP-4 inhibitors. It seems that SGLT2is are associated with a higher risk compared to GLP-1 receptor analogues but with a similar or lower risk compared to DPP-4 inhibitors [170,190,191,192,193,194].

Anyway, PAD itself is a risk factor for MALEs and limb amputation across all aforementioned groups of patients with type 2 diabetes, regardless of diabetic treatment mode [163,176]. However, when adding a new antidiabetic agent, we should also consider the risk of volume depletion. In patients treated with SGLT2is, volume depletion, a risk factor for acute limb ischemia, could be particularly dangerous in patients aged ≥ 75 y. compared to younger age categories [153,156,160,161,171].

The position of new antidiabetic agents in patients presenting with RAS and ICAS is unclear. From observational data and nephrologists’ opinions, the use of SGLT2is can contribute to a significant renal function deterioration in patients with RAS due to their mechanism of action [150,195]. However, it is not determined whether the use of GLP-1 receptor analogues and DDP-4 would be a better choice in patients with RAS, as adequate clinical studies are missing. Also, a relationship between stroke incidence and new glucose-lowering agents is debatable and, in particular, data in patients with ICAS are missing.

As diabetes contributes greatly to the development of polyvascular atherosclerotic occlusive disease, evolving additional mechanisms to those in lipid and blood pressure alterations, huge expectations are associated with new-generation antidiabetic medications. Adding a new glucose-lowering agent to the already used treatment gives hope for obtaining optimal glycemia control. A recent study in patients with RAS referred to renal artery angioplasty showed that maintained optimal diabetes control with a target glycated hemoglobin (HbA1C) below 7% (<53 mmol/mol) exerts a nephroprotective effect through the reduction of MACCEs and end-stage renal failure by 73% (95% CI = 0.13–0.57; *p* < 0.001) [143].

Last, but not least, sub-clinical chronic inflammation, endothelial dysfunction, and arterial stiffening that accelerates in aging patients impact outcomes in patients with polyvascular atherosclerotic disease [196,197,198,199]. So far, the pleiotropic anti-inflammatory, antithrombotic, and favorable metabolic effects were attributed to statin use [81]. Recently, several clinical studies are ongoing to elucidate the pleiotropic effects of new antidiabetic medications, namely GLP-1 receptor agonists and SGLT2is [172,198,200]. Research studies show favorable effects of SGLT2is on the telomere length, microRNA profile, pro- and anti-inflammatory cytokines, endothelial function, and arterial remodeling [201,202,203,204,205,206,207,208]. In line with this, similar research studies are ongoing with GLP-1 receptor agonists [209]. Thus, considerable emotions are associated with potential anti-inflammatory and antiatherothrombotic effects of GLP-1 receptor analogues and SGLT2is, though the current evidence on their efficacy on sub-clinical atherosclerosis, endothelial function, and arterial stiffness remains controversial.

## 6. Limitations

This study is not a systematic review, therefore the process of selecting the papers presented here may have been at risk of bias.

## 7. Conclusions and Future Directions

In summary, pharmacotherapy in atherosclerotic lesions outside coronary territory is similar but not identical to the prevention of CAD and mainly relies on the control of atherosclerotic risk factors. Some controversy surrounds antiplatelet and anticoagulant treatment in non-coronary artery atherosclerosis, depending on the course of asymptomatic stenosis or the presence of stenosis-related symptoms. The advanced age of patients demands a reduction in the number and doses of antithrombotic drugs to an absolute minimum. However, the role of statins cannot be underestimated in both primary and secondary prevention in patients with ICAS, PAD, and RAS, especially when diabetes is also present. In specific arterial areas, there may be a preference for certain drugs considering the elderly age of the patients. For example, statins are more effective at slowing down dementia when combined with ARBs, as well as nitrendipine. In the lower limb arteries, where the natural course of the disease depends both on PAD and diabetes management, statins play a crucial role; however, the role of novel diabetic agents needs to be elucidated in this area (limited evidence from EBM). We have also shown that well-balanced systolic and diastolic BP is very beneficial in atherosclerosis of non-coronary arteries, with optimal values of approximately 125/70 mmHg. Although these values may not be reasonable due to risk of orthostatic hypotension or frailty syndrome, they are beneficial for peripheral arteries and their vascularized organs. As we focus more on stabilizing atherosclerotic lesions and improving quality of life in the elderly and less on surgical and endovascular interventions for non-coronary artery stenosis, conservative management should be well planned in this patient population.

In the real world, two different attitudes can be observed. On the one hand, due to the known aging of the general population, more patients are on polytherapy and consequently exposed to side effects and drug interactions. Nevertheless, evidence suggests that prescriptions of a variety of therapeutic agents, including statins and novel lipid-lowering, antithrombotic, and antidiabetic medications are reasonable.

On the other hand, in the population of elderly patients, in particular those of 75 years and older, prescription of many drug classes is generally lower than in younger populations [210]. In particular, this regards use of statins and antiplatelet medications. Discontinuation of antiplatelet therapy can be observed in 33% of patients aged ≥ 65 years with newly diagnosed PAD, whereas after reinitiation only half remain on the therapy [211]. Data show that increasing age, history of ischemic stroke, and diabetes mellitus are associated with a decreased probability of discontinuation of antiplatelet treatment after reinitiation [211].

Often, clinical inertia of physicians hinders the review of therapy towards tailored therapy or deprescribing based on the clinical profile of the individual patient. The elderly are a heterogeneous age group in terms of functionality while being at the same time the most markedly growing age group in the world, especially in Europe.

Thus, in view of aging populations, with a life expectancy reaching 90, there is an urgent call for newly designed studies to provide evidence-based therapy.

## Figures and Tables

**Table 4:** Association between peripheral arterial disease and new glucose-lowering medications.

Study		Type of the Study (RCT, OS, Meta-Analysis	Study Design	Elderly Patients ≥75 y.o.	F-U Period	Main Findings	Outcomes	Remarks/Limitations
EMPEROR-Pooled Verma S et al., 2023 [137]	Empagliflozin 10 mg vs. placebo	RCT	In EMPEROR-Pooled (*n* = 9718, mean age 69 ± 10.5 y.), a total of 821 (8.4%) patients had PAD, mean age 72.2 ± 8.3 y.	NR	33 m.	In patients with HF (with either reduced or preserved ejection fraction), a significantly elevated risk of HF outcomes among patients with PAD compared with those without PAD was observed, including the composite of cardiovascular death and time to first HHF (HR, 1.51; 95% CI, 1.12–2.03; *p* = 0.007), time to CVD (HR, 1.40; 95% CI, 1.05–1.87; *p* = 0.02), time to all-cause mortality (HR, 1.42; 95% CI, 1.14–1.78; *p* = 0.002). Patients with PAD had a higher benefit for HHF absolute risk reduction	Patients with PAD are at higher absolute risk. Empagliflozin is efficacious in both PAD and no PAD groups. Patients with PAD had a higher absolute risk reduction in total HHF events compared with those without PAD (6.0% vs. 3.2%). There was no increase in adverse events with empagliflozin in patients with PAD, particularly rates of lower limb amputations	≥75 years: risk of volume depletion should be taken into account; ≥85 y.: not recommended
EMPA-REG OUTCOME Monteiro P et al., 2019 [140]	Empagliflozin vs. placebo		A total of 7020 patients with type 2 diabetes were treated, of whom 55.5%, 35.3%, and 9.3% were aged <65 y., 65–74 y., and ≥75 y.	N = 652 (9.5%)	Mean: 2 y.	The reductions in risk of CVD, HHF, HHF or CVD, and all-cause hospitalization with empagliflozin versus placebo in all three age categories were similar to the overall trial population (*p* = 0.484, *p* = 0.488, *p* = 0.240, and *p* = 0.638 for treatment-by-age group interaction, respectively)	The reductions in cardiovascular risk are consistent within all age categories. However, urinary tract infections (26.2% vs. 17.1%, *p* < 0.05) and volume depletion (6.8% vs. 4.9%, *p* < 0.05) were the most prevalent in patients aged ≥ 75 y. compared to younger age categories	No data on PAD and cardiovascular outcomes according to participants’ age; 25.4% of patients prematurely discontinued study medication
DECLARE-TIMI 58 Furtado RHM et al., 2019 [141]	Dapagliflozin vs. placebo	RCT	Diabetic patients with PAD, *n* = 1025 Diabetic patients without PAD, *n* = 16,135	6% of total study group	Median: 4.2 y.	Significant reduction in MACCEs, MALEs, and deaths, both in diabetic patients with PAD and without PAD		No safety data in severe liver disease
CANVAS Perkovic V et al., 2018 [144]	Canagliflozin 300 mg o.d. vs. placebo	RCT	A total of 10,142 patients with type 2 diabetes, mean age 63.3 y.	NR	Mean 2.4 y.	Canagliflozin use was associated with lower rate of MACCEs (occurring in 26.9 vs. 31.5 participants per 1000 patient-years; HR, 0.86; 95% CI, 0.75–0.97; *p* = 0.02 for superiority) and higher renal benefit (HR, 0.60; 95% CI, 0.47–0.78) compared with placebo	Increased rate of MALEs and amputations with canagliflozin (HR 1.97; 95% CI 1.41–2.75), particularly in patients > 65 years of age and with PAD. Eighteen more amputations per 10,000 people who received canagliflozin	Renal function and risk of volume depletion should be considered in patients above 65 y.o.
CREDENCE Perkovic V et al., 2019 [145]	Canagliflozin 100 mg o.d. vs. placebo	RCT	A total of 4401 patients with type 2 diabetes, mean age 63 y.	NR	Mean 2.5 y.	The canagliflozin group had a lower risk of MACCEs (HR, 0.80; 95% CI, 0.67–0.95; *p* = 0.01) and HHF (HR, 0.61; 95% CI, 0.47–0.80; *p* < 0.001) and lower risk of renal outcomes of end-stage kidney disease (HR, 0.70; 95% CI, 0.59–0.82; *p* = 0.00001)	There was no increased risk of amputation with canagliflozin (HR 1.11; 95% CI 0.79–1.56)	Renal function and risk of volume depletion should be considered in patients above 65 y.o.
SGLT2i (FLOZINS) EMPA-REG OUTCOME DECLARE-TIMI 58 Evans M et al., 2022 [146]	EMPA-REG: empagliflozin DECLARE-TIMI 58: dapagliflozin	Meta-analysis of RCTs	Diabetic and non-diabetic patients across age groups: <65 y., between 65–75 y., above 75 y. EMPA-REG: 35% of patients were 65–75 y.o., 9% were ≥75 y.o. DECLARE: 40% of patients were 65–75 y.o. and 6% ≥75 y.o.	9.3% in EMPA-REG trial 6% in DECLARE-TIMI trial	Mean: 2 y.	Patients aged < 65 y., 65–75 y., and ≥75 y. demonstrated similar reductions in risks of CVD, HF, and renal outcomes. The overall efficacy and safety of dapagliflozin and empagliflozin were also consistent regardless of age. It is recommended that before initiation of SGLT2i, patients should be on the optimal treatment with maximally tolerated RAA agent	SGLT2i should be avoided in hypotensive and hypovolemic patients. After SGLT2i initiation, there is a dip in baseline eGFR of 3 to 4 mL/min/1.73 m^2^. Repeat testing and close follow-up are recommended if eGFR declines by more than 20% to 25% with drug initiation, with dose reduction or discontinuation of therapy if eGFR drops by >30%	In patients with bilateral RAS or those with a single kidney, consultation with a nephrologist should be considered due to possible hemodynamic issues that may arise, especially when combined with a RAAS inhibitor Frailty was not considered
CANVAS CREDENCE Barraclough JY et al., 2022 [148]	Canagliflozin 100 mg or 300 mg o.d. vs. placebo	Meta-analysis of two RCTs	Of 14,543 participants (mean age 63 ± 8.5 y.), 3159 (21.7%) had PAD at baseline, mean age 63.8 ± 8.8 y.	NR	Mean: 2.5 y.	Patients with PAD are at higher absolute risk. The cumulative incidence of MACCEs, CVD or HHF, and all-cause mortality was substantially higher in patients with PAD compared with those without PAD. Canagliflozin reduced MACCEs (HR, 0.76; 95% CI, 0.62–0.92, *p* < 0.001)	There was no increase in the relative risk of extended MALEs with canagliflozin, irrespective of baseline PAD history (*p*-interaction > 0.864)	Relatively young age of study participants. No safety data in severe liver disease
SUSTAIN trial Cordiner R et al., 2016 [152]	Semaglutide *n* = 1648). Placebo *n* = 1649	RCT	A total of 3297 patients with type 2 diabetes, mean age 64.7 y.o.	NR	Median: 2.1 y	Semaglutide was associated with lower rates of MACCEs (HR, 0.74; 95% CI, 0.58 to 0.95; *p* < 0.001) and peripheral or coronary revascularization compared to placebo (HR, 0.65; 95% CI, 0.50–0.86; *p* = 0.003)	Semaglutide users had lower rates of MACCEs and MALEs. However, rates of retinopathy complications were higher in semaglutide users (HR, 1.76; 95% CI, 1.11 to 2.78; *p* = 0.02)	Relatively young age of study participants
Danish observational study Schäfer Z et al., 2023 [153]	GLP-1 analogues: liraglutide, dulaglutide, semaglutide, exenatide	OS	A total of 309,116 type 2 diabetes patients receiving GLP-1 analogues	≥70 y.: N = 74,339	Mean: 2.5 y.	Patients on GLP-1 treatment experience a notable reduction in the risk of amputation compared to those without the treatment with an HR of 0.5, 95% CI 0.54–0.74, *p* < 0.001	About 50% reduction of amputation risk	Use of GLP-1 increases the risk of gallbladder disease by 37% and the risk of nephrolithiasis by 27%
Scheen AJ et al., 2022 [154]	Any GLP-1 analogue vs. SGLT2i	Meta-analysis	A total of 505,355 patients on SGLT2is and 429,721 patients on GLP-1 analogue	NR	NR	The incidence rate was much higher in patients with cardiovascular disease or PAD (10.01 ± 1.07 in SGLT2i users vs. 7.66 ± 1.58 in GLP-1 analogue users, *p* = 0.0269) compared to patients without these complications (2.07 ± 0.71 in SGLT2i users vs. 1.48 ± 0.53 in GLP-1 analogue users, *p* = 0.0155)	In both groups, patients with PAD are at higher absolute risk for limb amputation. However, risk of limb amputation was significantly lower in the GLP-1 analogue group in comparison with the SGLT2i cohort (number of LLA events per 1000 patient-years: 3.54 vs. 4.72, *p* = 0.0043)	No data according to participants’ age
TECOS trial Bethel MA et al., 2017 [161]	Sitagliptin vs. placebo	RCT	Diabetic patients, *n* = 14,351	≥75 y.o. 2004 (*n* = 1979, 14%) patients	Median 2.9 y.	In the older cohort, sitagliptin did not significantly impact the composite (HR, 1.10; 95% CI, 0.89–1.36, *p* = 0.39), death (HR, 1.05; 95% CI, 0.83–1.32, *p* = 0.71), HF hospitalization (HR, 0.99; 95% CI, 0.65–1.49, *p* = 0.94), severe hypoglycemia (HR, 1.03; 95% CI, 0.62–1.71, *p* = 0.92) events	Rates of acute pancreatitis and pancreatic cancer or serious adverse events did not differ significantly in elderly vs. younger patients	Not reported: number of elderly patients with PAD and the results concerning PAD
SAVOR-TIMI 53 Trial Leiter LA et al., 2015 [162]	Saxagliptin vs. placebo	RCT	Elderly, *n* = 8561, and very elderly, *n* = 2330, patients with type 2 diabetes, within three age groups: 65–74, 75–84, and ≥85 y., initiating sitagliptin vs. non-DPP-4 inhibitor	*n* = 2330	Median: 2.1 y.	In patients ≥ 75 y. of age, treatment with saxagliptin compared to placebo showed no significant difference in the incidence of MACCEs (10% vs. 11.3%, *p* = 0.710), all-cause mortality (9.1% vs. 8.5%, *p* = 0.804), CVD (5.5% vs. 6.4%, *p* = 0.421), and MI (4.2% vs. 4.1%, *p* = 0.427). In saxagliptin group, there was significantly higher rate of HHF (6.8% vs. 4.9%, *p* = 0.026)	Treatment with saxagliptin in patients over 75 years of age is not associated with increased risk for adverse cardiovascular outcomes, except for HHF	No data on PAD and cardiovascular outcomes according to participants’ age
Chen DY et al., 2015 [165]	DPP-4 inhibitor (sitagliptin) vs. other diabetic medications	OS	A total of 5145 type 2 diabetic patients with recent ischemic stroke, mean age 67.6 ± 11 y., receiving sitagliptin (*n* = 1715), and 3430 patients (66.7%) who did not	≥75 y.: *n* = 1473	Mean: 1.17 y.	In patients ≥ 75 y.o., sitagliptin vs. other diabetic drugs showed no significant difference in the incidence of MACCEs and stroke compared to younger patients (12.1% vs. 11%, *p* = 0.463 and 8.6% vs. 8.1%, *p* = 0.705)	Treatment with sitagliptin in type 2 diabetic patients with recent ischemic stroke was not associated with increased or decreased risks of adverse vascular outcomes compared other diabetic agents	Similar incidence of acute and chronic pancreatitis in patients receiving sitagliptin and other diabetic agents (0.2% vs. 0.2%, *p* = 0.838) and the incidence of hypoglycemia (1.9% vs. 2.0%; *p* = 0.730)
SAVOR-TIMI 53 Trial Scirica BM et al., 2015 [166]	Saxagliptin vs. placebo	RCT	The effect of saxagliptin in 16,492 patients with T2DM, mean age 66 y., and a history of or at cardiovascular risk, including 11,423 (69.3%) with established arterial 1-bed disease; 1298 (7.9%) had 2-bed disease; and 104 (0.6%) had 3-bed disease	≥75 y.: *n* = 2330	Mean: 2.5 y.	Compared with diabetic patients with no established atherosclerosis, the adjusted HR for the MACCEs, in 1, 2, or 3 diseased beds, was 1.95, 3.54, and 4.64, respectively (trend *p* < 0.0001). The adjusted risk for overall mortality increased from 1.47 to 2.33 to 3.12, respectively (trend *p* = 0.0001). Saxagliptin use was not associated with increased risk of adverse outcomes compared placebo across each additional diseased arterial territory	Patients with PAD are at higher absolute risk. In patients with confirmed atherosclerosis, 8.5% had polyvascular disease; and compared with diabetic patients with single-bed disease, the risk of ischemic events and overall mortality was substantially higher in patients with polyvascular disease	Relatively young age of study participants

LAA—lower limb amputation, for other abbreviations please see Table 1 and Table 2.
